# Platelet-membrane-biomimetic nanoparticles for targeted antitumor drug delivery

**DOI:** 10.1186/s12951-019-0494-y

**Published:** 2019-05-13

**Authors:** Haijun Wang, Junzi Wu, Gareth R. Williams, Qing Fan, Shiwei Niu, Jianrong Wu, Xiaotian Xie, Li-Min Zhu

**Affiliations:** 1 College of Chemistry, Chemical Engineering and Biotechnology, Donghua University, Shanghai, 201620 China; 2 College of Basic Medicine, Yunnan University of Traditional Chinese Medicine, Kunming, 650500 China; 3 UCL School of Pharmacy, University College London, 29-39 Brunswick Square, London, WC1N 1AX UK; 4 Department of Pharmacy, Shandong Cancer Hospital Affiliated to Shandong University, Shandong Academy of Medical Science, Jinan, 250117 China

**Keywords:** Platelet membrane, Biomimetic, Bufalin, Nanoparticles, Targeted anti-tumor drug delivery

## Abstract

**Background:**

Nanoscale drug-delivery systems (DDSs) have great promise in tumor diagnosis and treatment. Platelet membrane (PLTM) biomimetic DDSs are expected to enhance retention in vivo and escape uptake by macrophages, as well as minimizing immunogenicity, attributing to the CD47 protein in PLTM sends “*don’t eat me*” signals to macrophages. In addition, P-selectin is overexpressed on the PLTM, which would allow a PLTM-biomimetic DDS to specifically bind to the CD44 receptors upregulated on the surface of cancer cells.

**Results:**

In this study, porous nanoparticles loaded with the anti-cancer drug bufalin (Bu) were prepared from a chitosan oligosaccharide (CS)-poly(lactic-co-glycolic acid) (PLGA) copolymer. These were subsequently coated with platelet membrane (PLTM) to form PLTM-CS-pPLGA/Bu NPs. The PLTM-CS-pPLGA/Bu NPs bear a particle size of ~ 192 nm, and present the same surface proteins as the PLTM. Confocal microscopy and flow cytometry results revealed a greater uptake of PLTM-CS-pPLGA/Bu NPs than uncoated CS-pPLGA/Bu NPs, as a result of the targeted binding of P-selectin on the surface of the PLTM to the CD44 receptors of H22 hepatoma cells. In vivo biodistribution studies in H22-tumor carrying mice revealed that the PLTM-CS-pPLGA NPs accumulated in the tumor, because of a combination of active targeting effect and the EPR effect. The PLTM-CS-pPLGA/Bu NPs led to more effective tumor growth inhibition over other bufalin formulations.

**Conclusions:**

Platelet membrane biomimetic nanoparticles played a promising targeted treatment of cancer with low side effect.

**Electronic supplementary material:**

The online version of this article (10.1186/s12951-019-0494-y) contains supplementary material, which is available to authorized users.

## Background

Nanoscale drug-delivery systems (DDSs) including inorganic nanoparticles [1, 2], liposomes [3, 4], hydrogels [5–7], and polymeric nanoparticles [8, 9] (among others) have great promise in tumor diagnosis and treatment. There are often problems with biocompatibility and short systemic circulation times when using these however, and much recent work has focused on using surface modifiers such as poly(ethylene glycol) (PEG) and proteins to ameliorate these issues [10–12]. Unfortunately, such PEG or protein modification can also impede the uptake of a DDS by cancer cells [13, 14]. Moreover, simple surface functionalization approaches are unable to precisely mimic the complex interfaces present in nature, and ultimately cannot completely vitiate recognition as a foreign body and the subsequent immune response [15, 16].

To overcome this hurdle to clinical translation, cell membrane cloaking has recently attracted significant attention as a route to endow nanoparticles with enhanced biocompatibility, low immunogenicity and active targeting abilities [17, 18]. Gao et al. [19] designed a set of nanoparticles (NPs) by encapsulating perfluorocarbon within the biocompatible polymer poly(lactic-co-glycolic acid) (PLGA), followed by coating with a red blood cell membrane (RBCM). These NPs were able to prolong the circulation time in the blood compared to uncoated particles, as well providing efficient intratumor penetration. The latter was attributed to the CD47 protein on the RBCM regulating phagocytic uptake by macrophages through interactions with SIRPα. In other work, Martinez et al. [20] designed leukocyte-based biomimetic nanoparticles as a novel theranostic platform for inflammatory diseases, and demonstrated that their materials have the ability to target the activated vasculature and exhibit superior accumulation in tumors and vascular lesions.

Platelets are components of the blood which exist to help prevent bleeding after blood vessel injuries. They avoid phagocytic uptake by macrophages, target and adhere to injured parts of the vasculature, and display enhanced binding to platelet-adhering pathogens [15]. Platelet membrane (PLTM) biomimetic DDSs thus are expected to enhance retention in vivo and escape uptake by macrophages, as well as minimizing immunogenicity as a result of the CD47 membrane protein sending “*don’t eat me*” signals to macrophages [21, 22]. In addition, P-selectin is overexpressed on the PLTM, which would allow a PLTM-biomimetic DDS to specifically bind to the CD44 receptors upregulated on the surface of cancer cells [23].

**Scheme 1:**
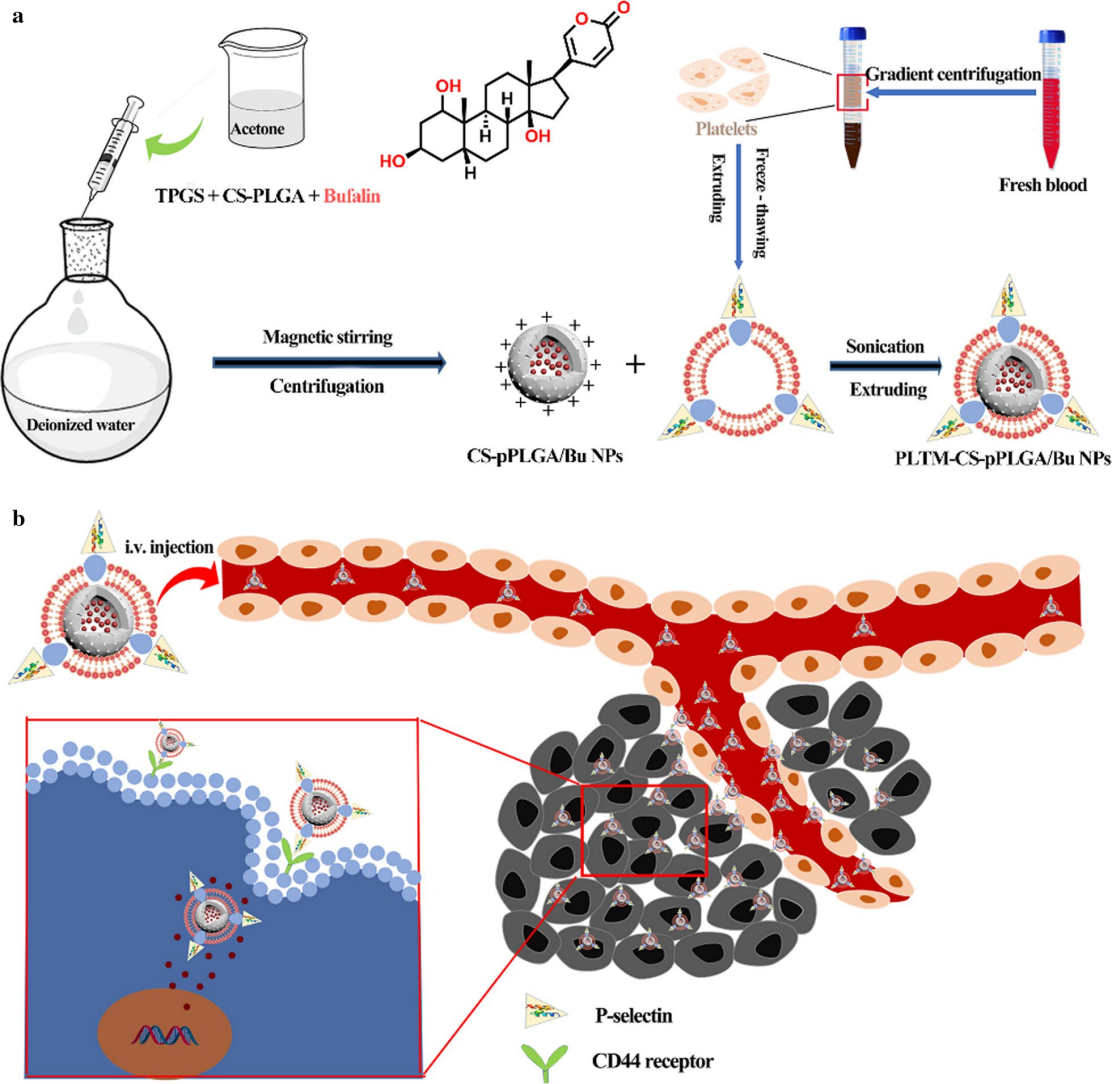
**a** Illustration of the preparation route to PLTM-CS-pPLGA/Bu NPs; **b** in vivo targeted bufalin delivery to a tumor site mediated by binding of P-selectin on the surface of the PLTM to CD44 receptors of the tumor cells

Here, we explored a strategy utilizing the PLTM to develop biomimetic nanoparticles with the aim of achieving enhanced biocompatibility and active cancer targeting. Poly(lactic-co-glycolic acid) (PLGA) nanoparticles were developed to deliver bufalin, an active ingredient which exhibits strong antineoplastic activities, including reversing multi-drug resistance, inhibiting cancer angiogenesis and regulating the immune response by inhibiting Na^+^, K^+^-ATPase [24, 25]. Moreover, bufalin is involved in complex cell-signal transduction pathways and results in selective control of tumor but not normal cellular proliferation [26, 27]. Typically, their long degradation times (weeks or even months) causes PLGA systems to release their drug cargo rather slowly, which limits the use of PLGA NPs in this setting [28]. However, the use of pore-forming agents such as Vitamin E polyethylene glycol succinate (TPGS) to prepare porous PLGA NPs can overcome this problem [29]. As displayed in Scheme 1, biodegradable PLGA was firstly conjugated to chitosan oligosaccharide (CS), followed by self-assembly with the porogen TPGS and bufalin by the nanoprecipitation method, resulting in porous bufalin-loaded nanoparticles (CS-pPLGA/Bu NPs) with positive surface charges. These were functionalized by absorbing the negatively charged PLTM by layer-by-layer (L-B-L) self-assembly, and the therapeutic effects of the NPs were explored in a murine tumor model.

## Materials and methods

### Materials

PLGA (lactide:glycolide 50:50, Mw ~ 21,000) was purchased from Jinan Daigang Biomaterials Co., Ltd. Chitosan oligosaccharide (pharmaceutical grade, Mw ~ 1200) was procured from Shanghai Xinxi Bio-Tech Co., Ltd, and bufalin from the Baoji Herbest Bio-Tech Co., Ltd. *N*-hydroxysuccinimide (NHS), 1-(3-dimethylaminopropyl)-3-ethylcarbodiimide hydrochloride (EDC·HCl), dicyclohexylcarbodiimide (DCC), fluoresceinisothiocyanate (FITC), Hoechst 33342, and 3-(4,5-dimethylthiazol-2-yl)-2,5-diphenyltetrazoliumbromide (MTT) were acquired from Sigma-Aldrich. Vitamin E TPGS and cyanine-5,5–carboxylic acid (Cy5.5–COOH, purity 99%) were obtained from J&K Scientific Ltd. H22 cells (a mouse hepatocellular carcinoma cell line) and RAW 264.7 (macrophage-like) cells were sourced from the Institute of Biochemistry and Cell Biology of the Chinese Academy of Sciences. A membrane protein extraction kit and protease inhibitor were procured from Phygene Life Sciences Co., Ltd. A Pierce BCA protein assay kit was procured from Life Technologies Co., Ltd. Mouse P-selectin protein (purity > 95%) was obtained from R&D Systems. Mouse anti-human CD41, mouse anti-human CD47, P-selectin antibody, DMEM medium, fetal bovine serum (FBS), phosphate buffered saline (PBS), penicillin and streptomycin were purchased from the Shanghai Bionove-gene Biomedical Technology Co., Ltd. All other chemicals were reagent grade and used as received.

### Synthesis of CS-PLGA copolymer

The CS conjugated PLGA copolymer was synthesized by an EDC coupling method. Briefly, PLGA (105 mg, 5 μmol) was dissolved in 10.0 mL of dimethyl formamide (DMF) and preactivated using EDC·HCl (2.9 mg, 15 μmol) and NHS (1.7 mg, 15 μmol) for 0.5 h in an ice bath. The temperature was allowed to rise to room temperature, after which 9 mg (7.5 μmol) of water-soluble CS was added and the reaction incubated under stirring for 48 h at room temperature, under an N_2_ stream. Any residual reactive agents and byproducts were removed by dialysis (MWCO 7000 Da) against deionized water for 2 days. The final product was collected after lyophilization, followed by ^1^H NMR analysis with a Bruker Advance 400 MHz spectrometer.

### Preparation and characterization of CS-pPLGA/Bu NPs

The preparation of bufalin-loaded porous CS-PLGA nanoparticles (CS-pPLGA/Bu NPs) was achieved following a literature nanoprecipitation method with some modifications [29]. 20 mg of CS-PLGA, 4 mg of TPGS and varied amounts of bufalin (CS-PLGA: bufalin ratio = 20:1, 10:1 or 5:1 w/w) were dissolved in 2 mL acetone, and the resultant solution then added dropwise to 10 mL of deionized water. The mixture was stirred at 1000 rpm in air for 3 h and then placed under vacuum for 12 h to evaporate any residual acetone. 2 mL of the resulting nanoparticles set aside for particle size and zeta potential measurements. The remainder of the dispersion was centrifuged and washed three times with deionized water to create porous nanoparticles. The supernatant was collected and tenfold diluted with methanol and a UV spectrophotometer at 298 nm (λ_max_ of bufalin) used to quantify the bufalin loading. The bufalin concentration was determined with reference to a pre-determined calibration curve. The bufalin encapsulation efficiency (EE %) and loading content (LC %) were determined as follows:

$${\text{EE}}\% = \frac{{{\text{mass}}\;{\text{of}}\;{\text{bufalin}}\;{\text{in}}\;{\text{NPs}}}}{{{\text{total}}\;{\text{mass}}\;{\text{of}}\;{\text{bufalin}}\;{\text{in}}\;{\text{feed}}}} \times 100\%$$

$${\text{LC}}\% = \frac{{{\text{mass}}\;{\text{of}}\; {\text{bufalin}}\;{\text{in}}\;{\text{NPs}}}}{{ {\text{total}}\;{\text{mass}}\;{\text{of}}\;{\text{NPs}}}} \times 100\% .$$

Bufalin-loaded non-porous CS-PLGA nanoparticles (CS-PLGA/Bu NPs) were prepared using the same method as above, except that TPGS was not added. Similarly, CS-pPLGA NPs without bufalin were generated following the same protocol but with no bufalin included in the preparation process. All the NP samples were stored at 4 °C until further use. For surface area and pore size measurements, a dry CS-pPLGA NPs powder were prepared after freeze drying. Measurements were performed on an automated area and pore size analyzer (Quantachrome, Autosorb-iQ).

### Preparation of PLTM

PLTM was obtained according to the manufacture’s instruction. The anti-coagulated whole blood of mice was added to centrifuge tubes above a separating medium layer (mice peripheral blood platelet separation medium, Tianjin Haoyang Bio-Tech Co., Ltd). The samples were centrifuged at 250–300 g for 15 min at room temperature. The supernatant comprised a platelet rich plasma (PRP). The PRP was diluted with an equal volume of sample diluent, followed by centrifuging at 500 g for 20 min. Platelets were obtained after washing the precipitate with phosphate buffered saline (PBS) buffer three times, and then resuspended in PBS containing 1 mM EDTA and 2 mM of protease inhibitor. The morphology of the platelets was assessed using an optical microscope (Nikon Eclipse E400). The platelet membrane was subsequently obtained by a freeze–thaw process assisted by sonication [15]. Platelet suspensions were frozen at − 80 °C before being thawed to room temperature; this process was repeated three times. Next, centrifugation at 4000 g for 3 min was employed to isolate the platelet membranes. The pellets were suspended in PBS buffer containing 2 mM of protease inhibitor and sonicated for 5 min at 40 kHz and a power of 100 W, resulting in a suspension of platelet membrane fragments.

### Preparation and characterization of PLTM biomimetic CS-pPLGA/Bu NPs

To coat PLTM onto CS-pPLGA/Bu NPs, 2 mL PBS containing 4 mg nanoparticles was mixed with PLTM fragments derived from 5 mL of whole blood. The mixture underwent sonication at 40 kHz and a power of 100 W for 5 min, followed by being extruded repeatedly (11 times) through a 200 nm porous membrane using a mini extruder (LiposoFast-Basic, Avanti). This resulted in PLTM-CS-pPLGA/Bu NPs. Zeta potential and particle size were measured at a concentration of 2 mg/mL by DLS (BI-200SM, Brookhaven Instruments), and the morphology was characterized using a transmission electron microscope (TEM; JEOL 2010F instrument). The size distribution of the CS-pPLGA/Bu NPs and PLTM-CS-pPLGA/Bu NPs was calculated from the analysis of around 100 nanoparticles in TEM images, using the Image J software. Blank PLTM-CS-pPLGA NPs were similarly prepared from the drug-free CS-pPLGA NPs.

### Western blotting

Western blotting was used to examine the platelet membrane proteins, following a reported procedure with some modifications [30]. Briefly, CS-pPLGA/Bu NPs and PLTM-CS-pPLGA/Bu NPs were normalized to equivalent overall protein concentrations using a Pierce BCA protein assay kit. The membrane proteins were extracted using a specific membrane protein extraction kit. 60 μL of the resultant buffered solution was used for sodium dodecyl sulfate-polyacrylamide gel electrophoresis (SDS-PAGE) on a 10% SDS-PAGE gel at 100 V, the proteins were then transferred using a semi-dry transfer method to a polyvinylidene difluoride (PVDF) membrane, followed by bathing in 5% skimmed milk (20 mL) for 2 h. Key platelet membrane proteins were identified using mouse anti-human CD41, mouse anti-human CD47, and P-selectin antibodies. The membranes were stored overnight at 4 °C and then washed with 50 mL of PBS containing 0.05% v/v Tween 20 before the addition of goat anti-rat HRP-conjugated secondary antibodies and incubation for another 2 h at room temperature. The membranes were combined with 1 mL of the ECL luminescent reagent followed by scanning (Epson Perfection V370 Photo, Seiko Epson Corporation) and imaging within the ImageJ software (National Institutes of Health) to quantify the amount of proteins present.

### In vitro drug release

The drug release profiles of CS-PLGA/Bu, CS-pPLGA/Bu and PLTM-CS-pPLGA/Bu NPs were evaluated by resuspending the NPs in PBS, and employing a dialysis method. 2 mL of a CS-pPLGA/Bu, CS-pPLGA/Bu or PLTM-CS-pPLGA/Bu NP water suspension (2 mg/mL) was loaded into a dialysis bag (MWCO = 7000 Da) and immersed in 18 mL of a release buffer (PBS containing 0.1% w/v Tween 80, pH 5.5 or 7.4). All experiments were performed at 37 °C with shaking (120 rpm) for 48 h. At predetermined time points, 1 mL of the external medium was collected and replaced with an equal volume of fresh pre-heated medium. The concentration of bufalin in the release medium was quantified by UV spectroscopy at 298 nm. Data are reported as mean ± standard deviation (SD), n = 3.

### MTT assays

The in vitro cytotoxicity of blank PLTM-CS-pPLGA NPs was studied to evaluate their cytocompatibility. H22 cells were maintained in DMEM medium supplemented with 1% penicillin, 1% streptomycin, and 10% v/v fetal bovine serum. The cells were cultured as a monolayer at 37 °C in a humidified atmosphere containing 5% CO_2_. For viability experiments, 200 μL of H22 cells were seeded into each well of 96-well plates at a density of 8 × 10^3^ cells/well. After overnight incubation at 37 °C in a humidified 5% CO_2_ environment, the medium was removed and 200 μL of fresh medium containing different concentrations of blank PLTM-CS-pPLGA NPs (1, 10, 25, 50, 100, 250 and 500 μg/mL) was added. After incubation for another 24 h, 20 μL of MTT solution (10 μg/mL) was added to the wells, and the plates incubated for an additional 4 h. After this, the medium was carefully removed, 200 μL of DMSO added to each well to dissolve the MTT-formazan generated by live cells, and the plate thoroughly shaken for 15 min. The absorbance of the wells was finally measured at 570 nm, using a microplate reader (Multiskan FC, Thermo Scientific).

The anticancer efficacy of the drug-loaded formulations was investigated following a similar protocol as described above, except that media containing free bufalin, CS-pPLGA/Bu NPs and PLTM-CS-pPLGA/Bu NPs (concentration of bufalin: 0.1, 0.5, 1, 5, 10, 20 μg/mL) was added after the initial incubation. Data are reported as mean ± SD, with three independent experiments each containing three replicates having been performed.

### Cellular uptake evaluation

In order to track the distribution of the PLTM-CS-pPLGA NPs visually in cells, we used FITC to label the CS-PLGA. Briefly, CS-PLGA (222 mg, 0.01 mmol) and FITC (19.5 mg, 0.05 mmol) were dissolved in 10 mL of dichloromethane, before being stirred for 12 h in the dark at 4 °C. The dichloromethane was removed by rotary evaporation, and 5 mL dimethyl formamide added to dissolve the product. This was followed by dialysis against 1 L of deionized water for 3 days (7000 Da Spectra/Por dialysis tube). Finally, the sample was freeze-dried for 48 h to obtain dry FITC labeled CS-PLGA. FITC labeled NPs (FITC-CS-pPLGA NPs and FITC-PLTM-CS-pPLGA NPs) were prepared using the same protocols as described above.

For cellular uptake evaluation, 200 µL of H22 cells or RAW 264.7 cells were seeded onto sterile coverslips placed in each well of a 24-well culture plate (5 × 10^4^ cells per well). 800 µL of DMEM supplemented with 1% penicillin, 1% streptomycin, and 10% v/v fetal bovine serum was added to each well. After incubation for 24 h, the medium was aspirated and replaced by 450 μL of fresh medium. 50 μL of PBS suspensions of FITC-CS-pPLGA or FITC-PLTM-CS-pPLGA NPs (1 mg/mL) were added. Following a further incubation step for 2 h (37 °C; 5% CO_2_), the culture medium was removed and the cells rinsed three times with PBS. They were then fixed with 4% paraformaldehyde for 20 min at 4 °C, and washed with PBS three times. The cell nuclei were stained with 0.5 mL of Hoechst 33342 (10 μg/mL) for 15 min at 37 °C and washed three times with PBS. Finally, the cells were studied using a confocal laser-scanning microscope (CLSM, Carl Zeiss LSM 700) with an argon blue laser light at 488 nm and a magnification of 63**×**. Each experiment was conducted in triplicate, and representative images are shown. For the blocking experiments, H22 cells were pre-incubated with free P-selectin (20 μg/mL) for 1 h. After removing the free P-selectin, the cells were incubated with FITC-PLTM-CS-pPLGA NPs (1 mg/mL) for another 2 h. The cells were then treated and imaged as described above.

### Flow cytometry

Flow cytometry was used to quantify NP uptake by H22 cells. 100 μL of H22 cells were cultured in six-well plates (1 **×** 10^6^ cells/well) for 24 h. 200 μL of PBS containing FITC-CS-pPLGA or FITC-PLTM-CS-pPLGA NP (1 mg/mL) were added to each well. After incubation at 37 °C for 4 h, the cells were digested by 0.25% w/v trypsin and 0.03% w/v EDTA and centrifuged at 1000 rpm for 5 min. The supernatant culture medium was discarded, and the precipitate dispersed in 500 μL of PBS and incubated for 15 min in the dark. Finally, cellular uptake was determined by flow cytometry using a Becton–Dickinson FACScan analyzer (Frankin, CA). A blocking experiment was also performed to evaluate the active targeting effect mediated by selective blinding between P-selectin and the CD44 receptor. H22 cells were first pre-incubated with free P-selectin (20 μg/mL) for 1 h, after which the free P-selectin was removed and the cells incubated with FITC-PLTM-CS-pPLGA NPs (1 mg/mL) for another 4 h at 37 °C. Cellular uptake was then probed by flow cytometry as detailed above.

### Hemocompatibility assay

Fresh blood was obtained from a healthy ICR mouse and centrifugation performed at 3000 rpm for 5 min to obtain red blood cells (RBCs). The RBCs were washed three times using PBS (pH 7.4), and then treated with equal amounts of PBS (negative control), Triton X-100 (positive control) and a 500 μg/mL suspension of PLTM-CS-pPLGA NPs in PBS. The samples were incubated at 37 °C for 1 h and centrifugation was performed at 5000 rpm for 10 min. 100 μL of the supernatant from each tube carefully collected and added to each well of 96-well plates in triplicate. The absorbance of each well was obtained at 540 nm in a microplate reader (Multiskan FC, Thermo Scientific) and the formula below used to calculate the percentage of RBC hemolysis [31]. The whole procedure was performed three times.

$$Hemolysis \;\% \, = \,OD\left[ {\text{test}} \right] - OD [negative \, control]\; \times 100/OD [positive \, control]$$

### Tumor model mice

All animal experiments were undertaken following robust ethical review and in accordance with the procedures authorized by the Committee for Experimental Animal Welfare and Ethics of Yunnan University of Traditional Chinese Medicine. ICR female mice (specific pathogen-free grade, 18–20 g) were procured from Jiangsu KeyGENBioTECH Co. Ltd, and tumors implanted by subcutaneous injection of 1 × 10^6^ H22 cells in 100 μL of PBS (pH = 7.2–7.4) into the right front limb armpit of each mouse. Tumor volume was calculated as width^2^ × length/2.

### In vivo and ex vivo imaging

In vivo fluorescence imaging was performed using an IVIS Lumina LT imaging system (Caliper Life Sciences) to investigate the biodistribution of Cy5.5 labeled PLTM-CS-pPLGA NPs. The latter were prepared according to the literature [32], with some modifications. 6 mg of Cy5.5–COOH was dissolved in 1 mL DCM containing 2.2 mg DCC, and placed for 30 min in an ice bath. 6.5 mL of a DCM solution containing 320 mg CS-PLGA and 1.3 mg DMAP was then added into the above solution, and the two allowed to react under gentle stirring in the dark overnight. Next, the solvent was evaporated using a rotary evaporator. Unreacted Cy5.5–COOH was removed by washing with ice-cold methanol and recovery of the solid product by centrifugation at 5000 rpm. The washing process was repeated three times, resulting in Cy5.5-CS-PLGA. This was then processed into nanoparticles using the same procedures as described above.

200 μL of Cy5.5 labeled bufalin-free PLTM-CS-pPLGA NPs or Cy5.5 labeled CS-pPLGA NPs in PBS (2 mg/mL) were i.v. injected into each mouse when the tumor volume reached 200 mm^3^. Fluorescent imaging at various time intervals after the injection was performed using a 60 s exposure time, with excitation at 748 nm and emission at 780 nm. For ex vivo imaging, H22 tumor-bearing mice were sacrificed by cervical vertebra dislocation 6 h after i.v. injection with the Cy5.5 labeled NPs. The tumor and major organs were collected and imaged using the IVIS Lumina LT imaging system.

### Immunofluorescence assay

To further evaluate the targeted effect of PLTM biomimetic NPs to tumor through recognizing CD44 receptor on tumor cells, tumors from mice after treating PLTM coated or uncoated Cy5.5 labeled CS-pPLGA/Bu NPs for 24 h were sectioned into 100 μm sections under frozen conditions. The tumor tissues were fixated with paraformaldehyde, and then incubated with primary antibody (rat anti-mouse CD44, Servicebio) against CD44 at 4 °C overnight followed by the incubation with secondary antibody (goat anti-rat IgG, DyLight 488, Servicebio) at 37 °C for 50 min. The nuclei of the cells were stained with DAPI. The obtained slices were scanned by an imaging system (Nikon DS-U3).

### In vivo antitumor efficacy

Mice bearing H22 tumors were treated after their tumors reached approximately 120 mm^3^ in size. The mice were randomly divided into four groups (5 mice per group) and given 200 μL of PBS, free bufalin, CS-pPLGA/Bu NPs, or PLTM-CS-pPLGA/Bu NPs (bufalin dose: 2 mg/kg) intravenously every 2 days. Tumor sizes and body weights were monitored and recorded every 2 days for 16 days, after which the experiment was halted. After this time, the mice were sacrificed by cervical vertebra dislocation. The tumors and major organs of each group were extracted. The tissues were fixed with 4% paraformaldehyde solution and embedded in paraffin. 4 mm slices were taken, mounted on glass slides, stained by hematoxylin and eosin (H&E), and imaged with a digital microscope (Nikon DS-U3 microscope). The tumors tissues were further stained with terminal deoxynucleotidyl transferase UTP nick end labeling (TUNEL) to study the extent of apoptosis in the tumors.

### Toxicity

In order to exclude PLTM effects on tumor growth inhibition and evaluate the biosafety after the administration of PLTM-CS-pPLGA NPs, mice bearing H22 tumors (~ 120 mm^3^ in size) were randomly divided into two groups (5 mice per group) and given 200 μL of PBS or a suspension of the PLTM-CS-pPLGA NPs (at the same dose as the therapeutic group) intravenously every 2 days. Tumor sizes were monitored and recorded every 2 days for 16 days. On day 16, 0.5 mL of blood was withdrawn for blood biochemistry analysis. The blood samples were centrifuged at 3500 rpm for 15 min at 4 °C to obtain serum. The serum concentrations of alanine aminotransferase (ALT), aspartate aminotransferase (AST), creatinine (CREA) and urea nitrogen (BUN) were then analyzed using an automatic biochemistry analyzer (7600, Hitachi). The liver and kidney were removed for histological assessment by H&E staining.

### Statistical analysis

Differences between two groups were analyzed by Student’s *t* test and mean values were compared via one-way ANOVA; The significance level was defined as P < 0.05, and the data were marked with (*) P < 0.05, (**) P < 0.005 and (***) P < 0.001.

## Results and discussion

### Synthesis and characterization of CS-PLGA

In order to construct nanoparticles with a positive surface charge for absorbing negative charged PLTM by L-B-L assembly, a water-soluble chitosan oligosaccharide was initially conjugated to PLGA. ^1^H-NMR spectra (Additional file 1: Figure S1) showed that a new resonance peaks at δ = 7.9(m) appeared after the conjugation. This can be ascribed to the amide bond (–CO–N**H**–). All other hydrogen signals from CS and PLGA are also visible in the CS-PLGA spectrum, demonstrating successful conjugation. Zeta potential data confirmed this. When the carrier phase pH was adjusted from 8.6 to 5.0, the zeta potential of CS-PLGA went from a negative to a positive value (Additional file 1: Figure S2), ascribed to the increased protonation of the CS segments under acidic conditions. In contrast, the zeta potential of CS and PLGA show much more modest changes in response to pH variation; CS has an increasingly negative zeta potential as the pH goes up, while PLGA becomes less positively charged.

### Preparation of PLTM-coated biomimetic NPs

**Fig. 1:**
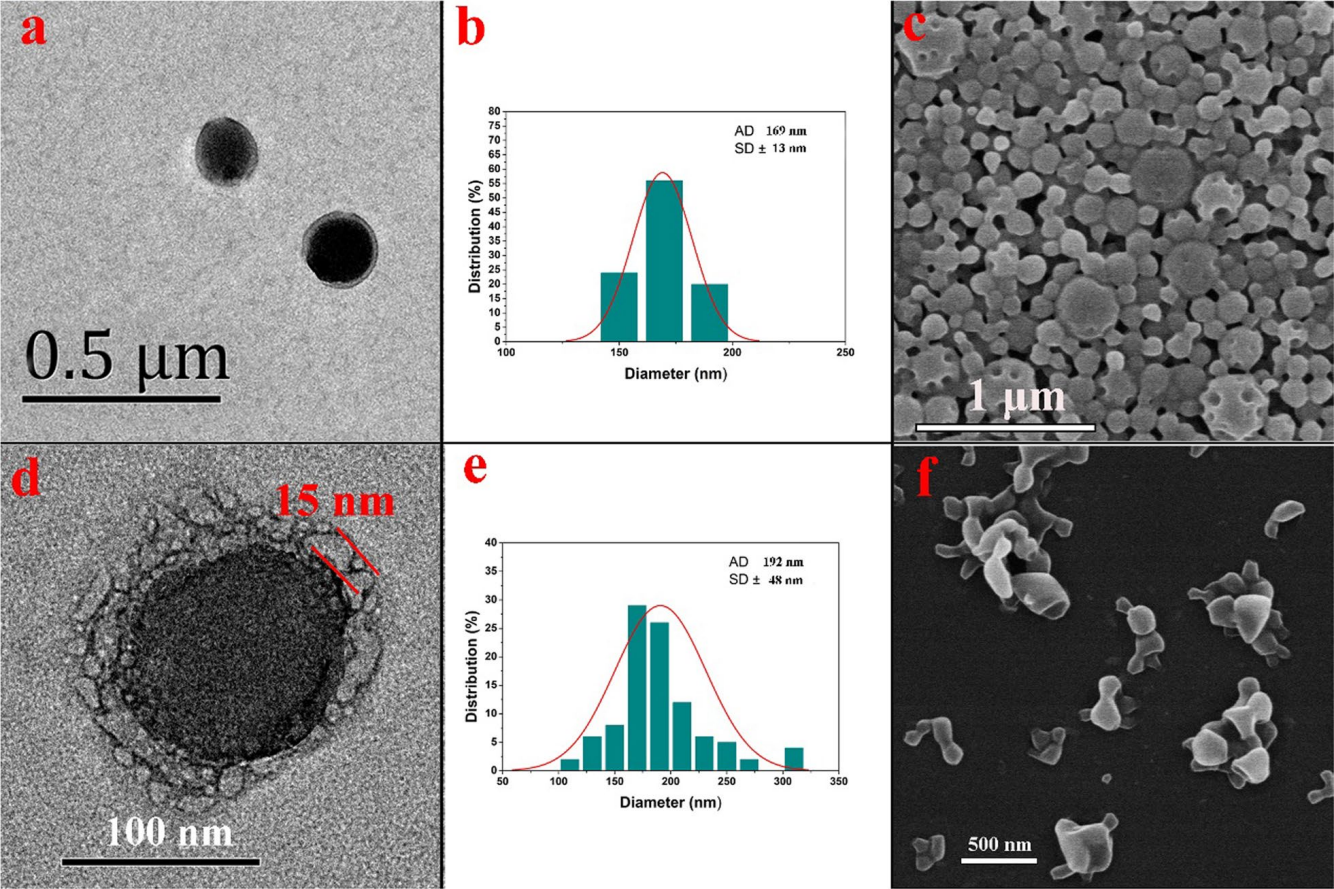
Characterization date on the CS-pPLGA/Bu NPs: **a** a TEM image, **b** the particle size distribution and **c** a FESEM image, and the PLTM-CS-pPLGA/Bu NPs: **d** a TEM image, **e** the particle size distribution and **f** a FESEM image

The pore forming agent TPGS was used generate porous CS-PLGA particles. TEM images (Fig. 1a) show that CS-PLGA/Bu NPs show a core/shell morphology. The core manifests as a dark area surrounded by a lighter grey CS shell, and the particles have a mean size of ~ 170 nm (Fig. 1b). The small size of these particles means that the porosity cannot be seen. In order to ensure that the TPGS did indeed generate pores in the formulations, larger particles were prepared with a low stirring speed; surface pores can be clearly observed in these (Fig. 1c), demonstrating that TPGS can serve as a pore-forming agent. Brunauer–Emmett–Teller (BET) measurements showed that the surface area and average pore diameter of CS-pPLGA NPs were 11.65 m^2^ g^−1^ and 3.05 nm (Additional file 1: Figure S3), further confirming the porosity of the nanoparticles.

Platelets (PLTs) were successfully extracted from fresh whole blood (Additional file 1: Figure S4) by gradient centrifugation, and then subjected to repeated freeze–thaw process to obtain the empty platelets. After PLTM coating by sonicating and extruding the mixture of CS-pPLGA NPs and empty platelets, a cell-membrane structure can be clearly observed around the CS-pPLGA NPs, with an outer shell ≈ 15 nm in thickness (Fig. 1d), and the particles have a mean size of ~ 192 nm (Fig. 1e). FESEM images of PLTM-CS-pPLGA NPs (Fig. 1f) also demonstrate the nanoparticles to be wrapped by an external layer. The zeta potential of the nanoparticles after PLTM coating decreased to ~ − 28 mV (cf. ~ − 5 mV for CS-PLGA; Additional file 1: Figure S5); the PLTM-CS-pPLGA NPs thus possess the same surface charge as the PLTM, consistent with the existence of the latter on the surface of the NPs.

**Fig. 2:**
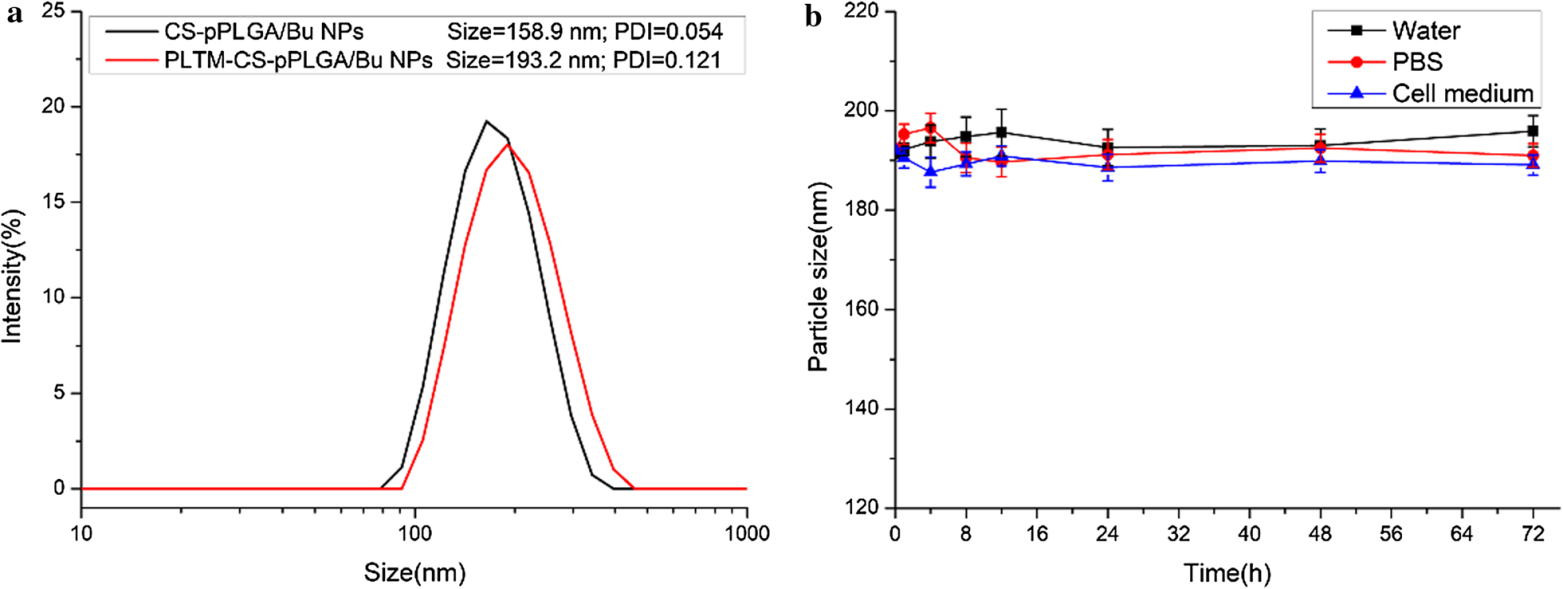
DLS sizes of **a** CS-pPLGA/Bu NPs and PLTM-CS-pPLGA/Bu NPs in water; **b** PLTM-CS-pPLGA/Bu NPs in different solutions, including water, phosphate buffered saline (PBS) and 10% v/v serum-containing DMEM cell culture medium. Data are presented as mean ± SD (n = 3)

The hydrodynamic particle size of the two NPs and the colloid stability of the PLTM-CS-pPLGA/Bu NPs in water, PBS and cell culture medium were quantified by DLS. The results are presented in Fig. 2. The mean size of all the NPs lies between 150 and 195 nm, and their polydispersities are low (Fig. 2a). The coated PLTM-CS-pPLGA/Bu NPs exhibited high colloidal stability and could be dispersed in different physiological buffers without aggregation over time (Fig. 2b); this is attributed to the stabilizing effect played by the plasma membrane’s hydrophilic surface glycans [33].

### Platelet membrane proteins

The presence of key platelet membrane proteins, including the platelet-specific CD41, immunomodulatory CD47, and the cancer-targeted P-selectin on the nanoparticles was examined by Western blotting. It was observed that the PLTM-CS-pPLGA NPs have an exterior protein environment very similar to the platelet membrane source (Additional file 1: Figure S6).

### Drug loading and release behavior

The results of encapsulation efficiency and loading content of the bufalin-loaded porous CS-pPLGA NPs are summarized in Additional file 1: Table S1. Although, the highest LC (11.5 ± 2.1%) was achieved when the mass ratio of Bu: CS-PLGA was 1:5, the EE was only 65.4 ± 4.2%, leading to wastage of bufalin. When the ratio reached to 1:20, the low LC (only 4.6 ± 1.3%) rendered the NPs non-viable for further exploitation. The 1:10 ratio offers a good balance between these two extremes, with an LC and EE of 8.5 ± 1.9% and 92.8 ± 3.5%, respectively.

**Fig. 3:**
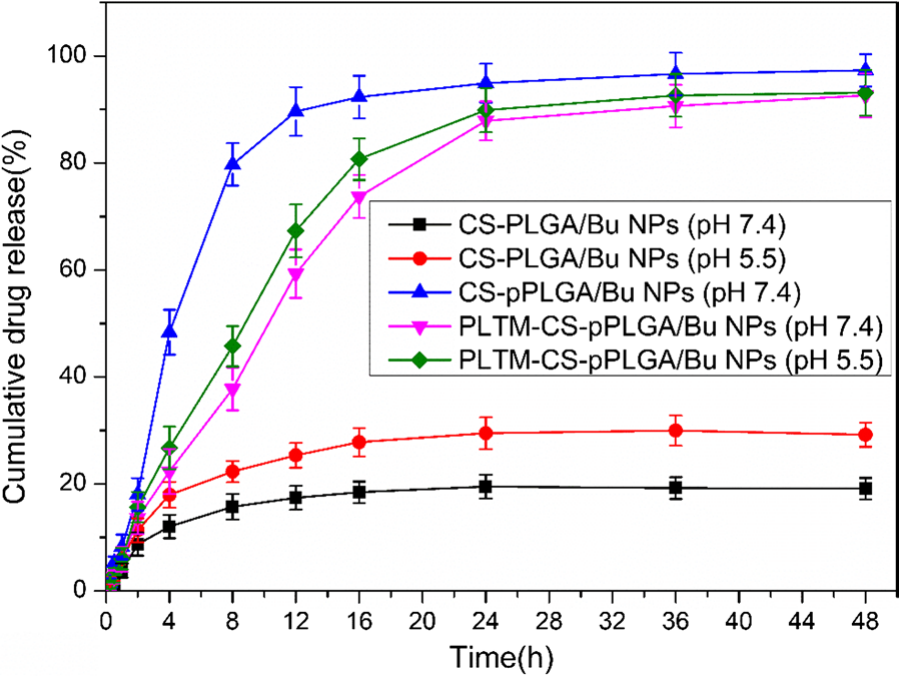
In vitro drug release profiles of CS-PLGA, CS-pPLGA, and PLTM-CS-pPLGA NPs. Data are presented as mean ± SD (n = 3)

Drug release data in PBS at pH 7.4 and pH 5.5 (simulating the slightly acidic tumor pH) are given in Fig. 3. At pH 7.4, only around ~ 20% of the bufalin cargo is released from the non-porous CS-PLGA NPs over 48 h. In contrast, the CS-pPLGA NPs gave a burst of drug release in the initial 4 h of the experiment, and reached a cumulative release of 90.6 ± 4.5% after 12 h. This can be attributed to their porous structure. This burst release was inhibited after coating with the PLTM, which can be ascribed to the compact lipid bilayer at the exterior of the particles blocking bufalin release. The release of bufalin was much faster in the simulated acidic tumor environment (pH 5.5) than at pH 7.4. This is likely to be because the CS segments are more soluble under acidic conditions, freeing their drug cargo as they dissolve [34]. Overall, it is clear that the PLTM-CS-pPLGA NPs can provide pH-dependent sustained release, and thus are potentially useful in the treatment of cancer.

### MTT assay

**Fig. 4:**
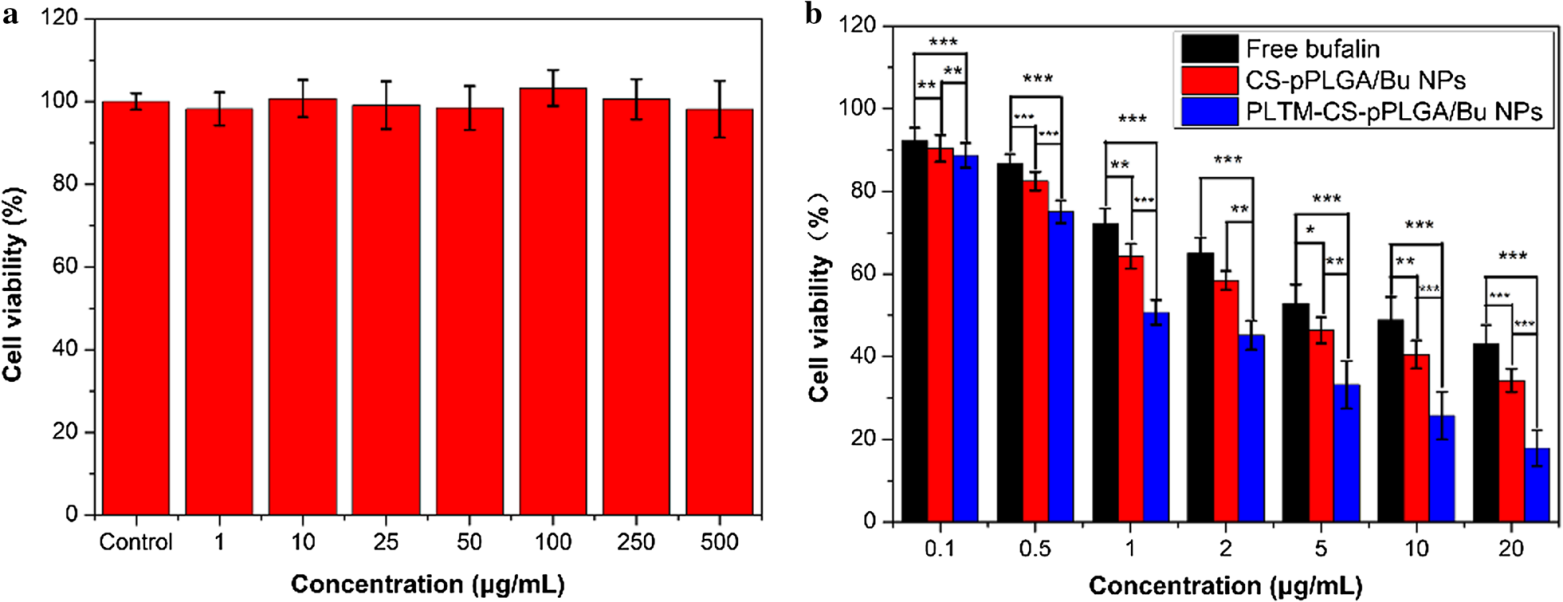
MTT viability results for **a** H22 cells treated with blank PLTM-CS-pPLGA NPs, **b** H22 cells treated with free bufalin, CS-pPLGA/Bu and PLTM-CS-pPLGA/Bu NPs

The cytocompatibility of the blank PLTM-CS-pPLGA NPs with H22 cells was investigated using the MTT assay (Fig. 4a). No in vitro toxicity was observed for the nanoparticles even when the concentration reached 500 μg/mL. The carrier can thus be regarded as having good cytocompatibility. The PLTM-CS-pPLGA/Bu NPs have clear toxicity to H22 cells (Fig. 4b), and are more potent than free bufalin in this regard. When the bufalin concentration is raised to 20 μg/mL, the cell viability declines to 43.0%, 34.2% and 17.9% with free bufalin, the CS-pPLGA/Bu NPs and PLTM-CS pPLGA NPs, respectively. Similar trends are seen at the other concentrations studied: the PLTM coating clearly enhances the efficacy of the formulation. This may be attributed to efficient internalization mediated by the targeted binding of P-selectin on the surface of PLTM to CD44 receptors of H22 cells.

### Cellular uptake of nanoparticles

**Fig. 5:**
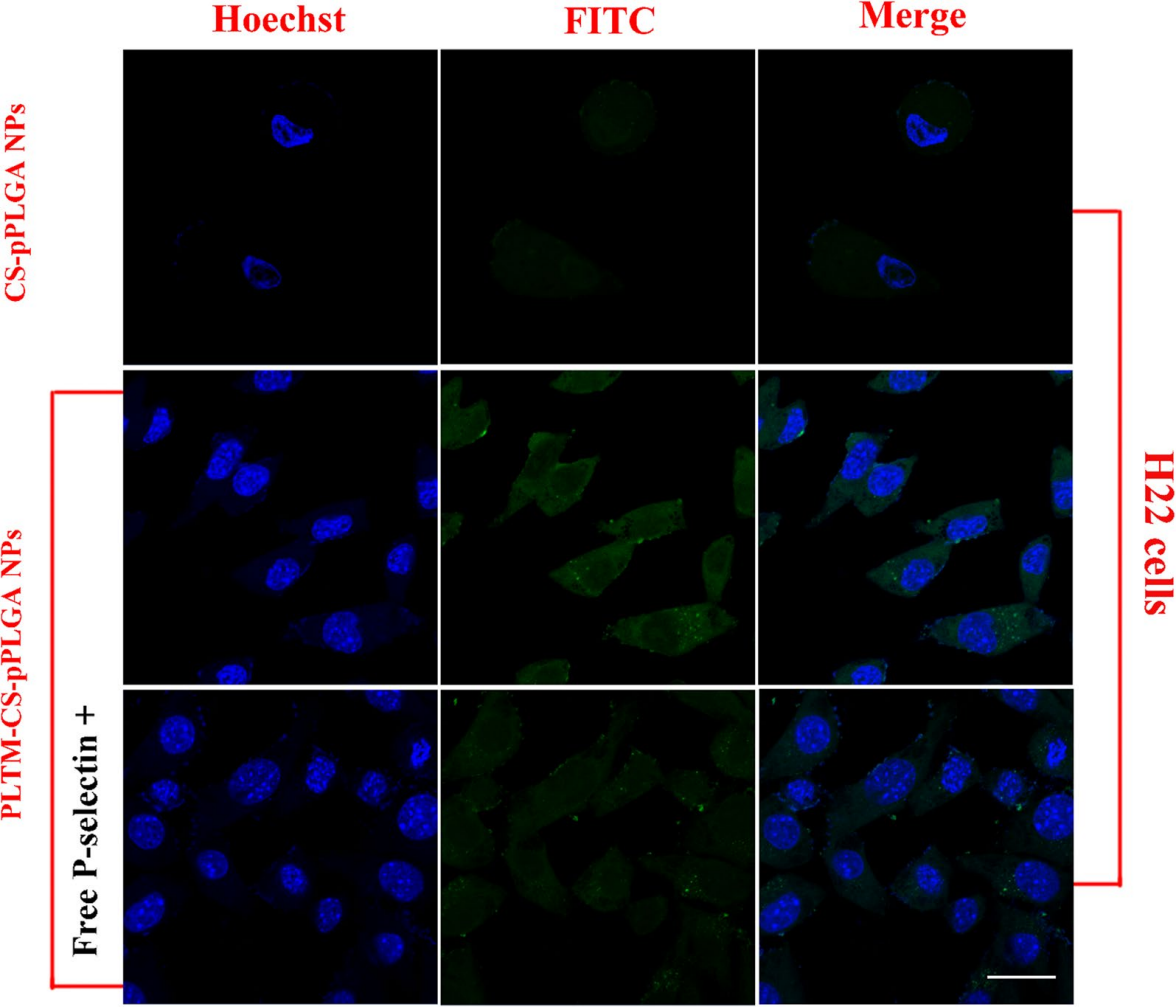
CLSM images of H22 cells following 2 h incubation with FITC-CS-pPLGA, FITC-PLTM-CS-pPLGA NPs, after a 1 h pre-treatment with or without free P-selectin. Scale bar: 20 μm

**Fig. 6:**
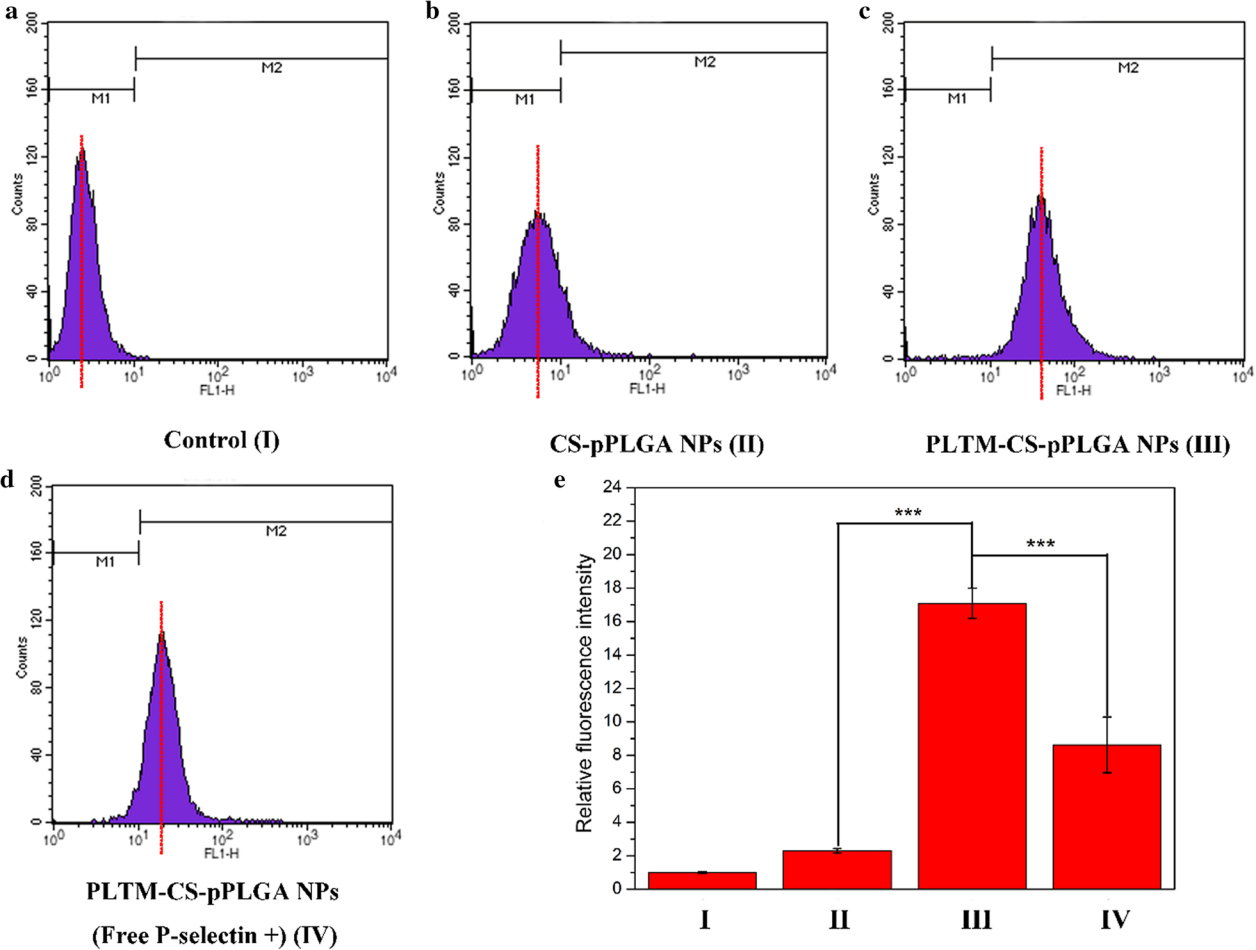
Flow cytometry data for: **a** untreated H22 cells; H22 cells incubated for 4 h with FITC-labeled **b** CS-pPLGA NPs or **c** PLTM-CS-pPLGA NPs; and, **d** cells pre-incubated with P-selectin for 1 h before being exposed to FITC-PLTM-CS-pPLGA NPs for 4 h, and **e** corresponding quantified fluorescence intensity

The cellular uptake of FITC-CS-pPLGA or FITC-PLTM-CS-pPLGA NPs was probed by CLSM. As shown in Fig. 5, H22 cells treated with FITC labeled PLTM-CS-pPLGA NPs exhibited strong intracellular fluorescence intensity. In contrast, only weak fluorescence intensity localized inside or around the cellular membranes was seen when H22 cells were treated with FITC-CS-pPLGA NPs: the NPs appeared unable to reach the nuclei of H22 cells. To further evaluate the endocytosis mechanism of cellular uptake, a blocking experiment was performed in which the H22 cells were pre-treated with free P-selectin. CLSM (Fig. 5) showed that the fluorescence signals in free P-selectin pre-incubated H22 cells were significantly reduced. In accordance, flow cytometry (Fig. 6) demonstrated that the cellular uptake of FITC-PLTM-CS-pPLGA NPs by H22 cells was about 7.4-fold greater than uptake of FITC-CS-pPLGA NPs. The cellular uptake was, however, significantly reduced by pre-incubated H22 cells with free P-selectin. These results indicate that the biomimetic membrane can enhance internalization by cancerous cells, presumably attributed to the binding of P-selectin on the PLTM to CD44 receptors of H22 cells.

The CLSM results for RAW 264.7 cells treated with FITC-PLTM-CS-pPLGA NPs are rather different to those obtained with H22 cells (Additional file 1: Figure S7). The NPs are mainly located around the cell membranes, demonstrating that the biomimetic nanoparticles contained “self-recognized” proteins (CD47). These inherently mimic the surface properties of the host cells and thus can escape uptake by macrophages and send “*don’t eat me*” signals to the host [22].

### Hemocompatibility assay

A hemolysis assay was performed to evaluate the biocompatibility of PLTM-CS-pPLGA NPs with RBCs. Microscopic observations (Additional file 1: Figure S8) showed that no morphological changes and no significant lysis were observed after RBCs were treated with a high concentration of PLTM-CS-pPLGA NPs (500 μg/mL). The amount of hemoglobin released from RBCs into solution permits the degree of hemolysis to be quantified: spectroscopic results showed only ~ 3.85% RBC lysis (Additional file 1: Table S2). The results indicated that the PLTM-CS-pPLGA NPs are blood-compatible biomaterials according to the ISO/TR 7406 standard [35].

### In vivo biodistribution in H22 tumor-bearing mice

**Fig. 7:**
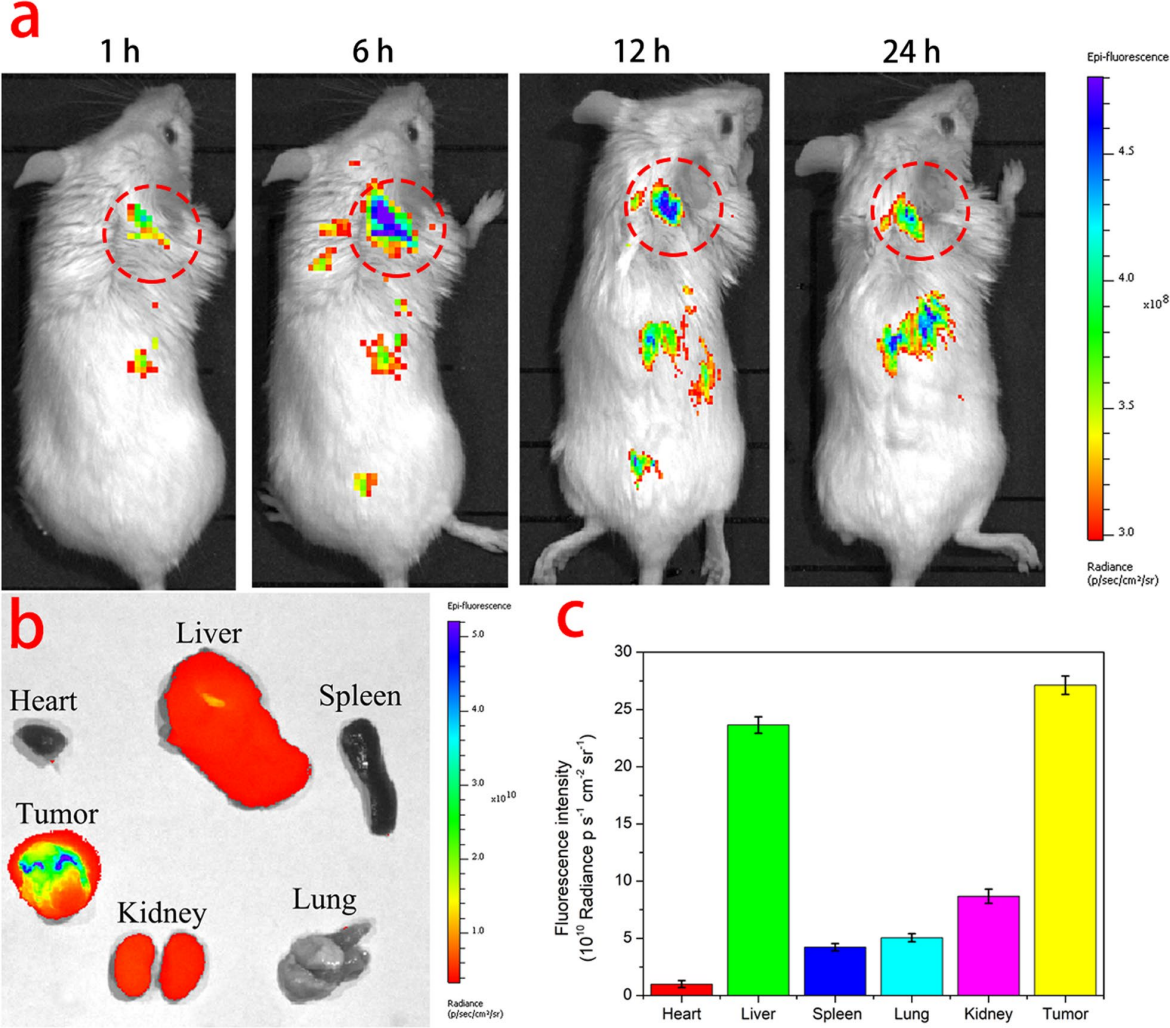
**a** Time-dependent in vivo fluorescence imaging of Cy5.5 labeled PLTM-CS-pPLGA NPs in H22 tumor-bearing ICR mice. **b** Ex vivo fluorescence imaging of the excised tumors and normal organs at 6 h post-injection. **c** ROI analysis of fluorescent intensities from the tumor and major organs. Error bars indicate SD (n = 3)

In vivo and ex vivo fluorescence imaging was used to evaluate the tumor targeting capability of Cy5.5 labeled PLTM-CS-pPLGA NPs and Cy5.5 labeled CS-pPLGA NPs. Strong fluorescence signals were observed at the tumor site 6 h after injection of the PLTM-CS-pPLGA NPs (Fig. 7a), demonstrating the targeting ability of the biomimetic nanoparticles. Elevated fluorescence intensity was also found at the tumor site even at 24 h post injection. After 6 h imaging, one mouse was sacrificed and the tumor and major organs harvested for ex vivo imaging. The fluorescence intensity at the tumor site was much higher than in the other organs (Fig. 7b), indicating that PLTM-CS-pPLGA NPs have good tumor selectivity. The results were confirmed by quantitative region-of-interest (ROI) analysis (Fig. 7c), which showed 1.15-fold and 3.12-fold high fluorescence intensity in the tumor than in the liver and kidney respectively.

In contrast, low fluorescence signals were observed at the tumor site for the uncoated Cy5.5 labeled CS-pPLGA NPs during whole treatment time (Additional file 1: Figure S9a). Ex vivo imaging results (Additional file 1: Figure S9b) showed weaker fluorescence signals in the tumor tissue, but stronger fluorescence in the liver and kidney. Quantitative ROI analysis result (Additional file 1: Figure S9c) showed the fluorescence intensity in the liver and kidney were 6.2-fold and 3.2-fold higher than that in tumor respectively. Collectively, these in vivo findings confirm that the nanoparticles coated with the PLTM could inhibit uptake by the reticuloendothelial system and achieve active targeting to the tumor.

### Immunofluorescence assay

**Fig. 8:**
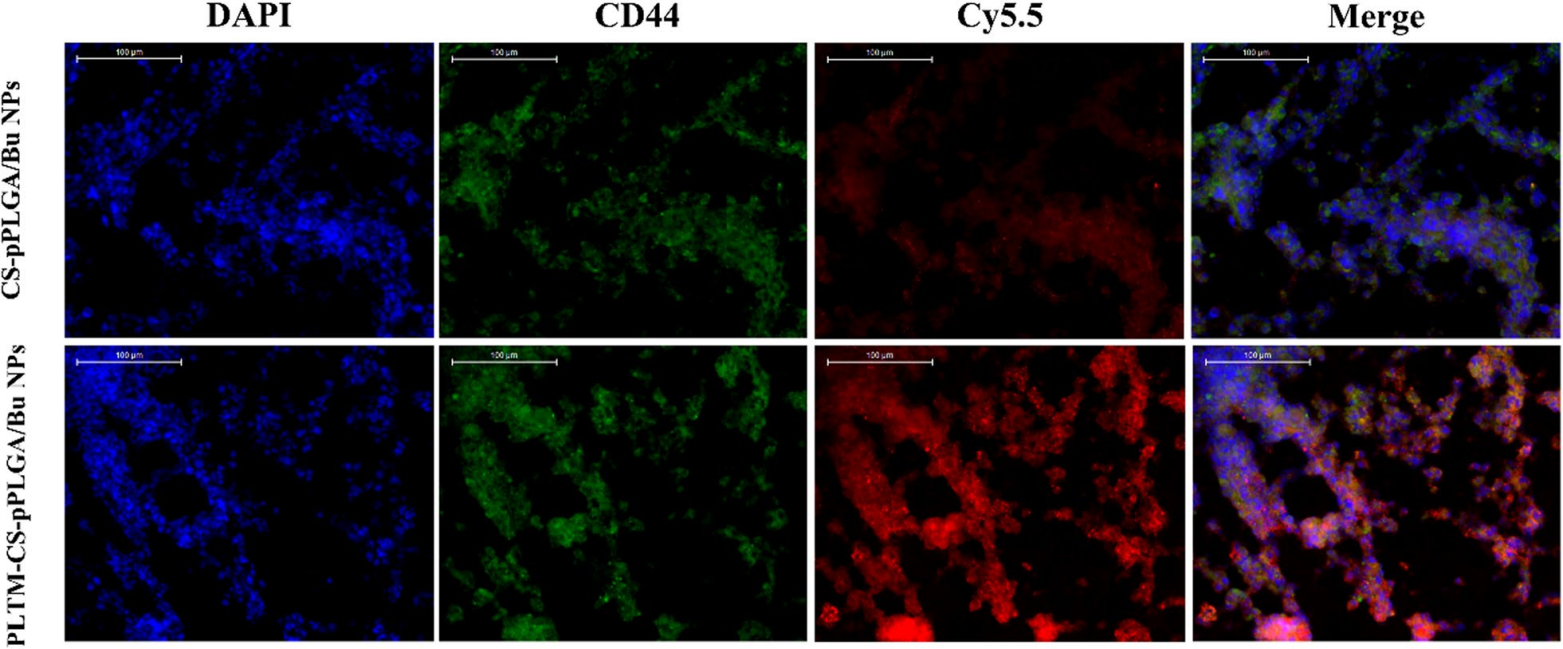
Localization of NPs in H22 bearing tumor tissues. Mice bearing H22 tumor were intravenously injected with Cy5.5-labeled CS-pPLGA/Bu NPs and Cy5.5-labeled PLTM-CS-pPLGA/Bu NPs. 24 h later, the tumors were removed and sectioned under frozen conditions followed by staining with an antibody against CD44 (green). The nuclei of cells were visualized using DAPI (blue)

Immunofluorescence assay was subjected to evaluate the targeting ability of PLTM biomimetic NPs on the histological level. As shown in Fig. 8, strong red fluorescence of Cy5.5-labeled PLTM-CS-pPLGA/Bu NPs could be observed in tumor tissues, while tumor tissues treated with Cy5.5-labeled CS-pPLGA/Bu NPs exhibited weak red fluorescence. The results demonstrated that PLTM would drive the NPs to specifically bind to tumor cells, which attributing to the binding of P-selectin on the PLTM to CD44 receptors of H22 cells.

### In vivo antitumor efficacy

**Fig. 9:**
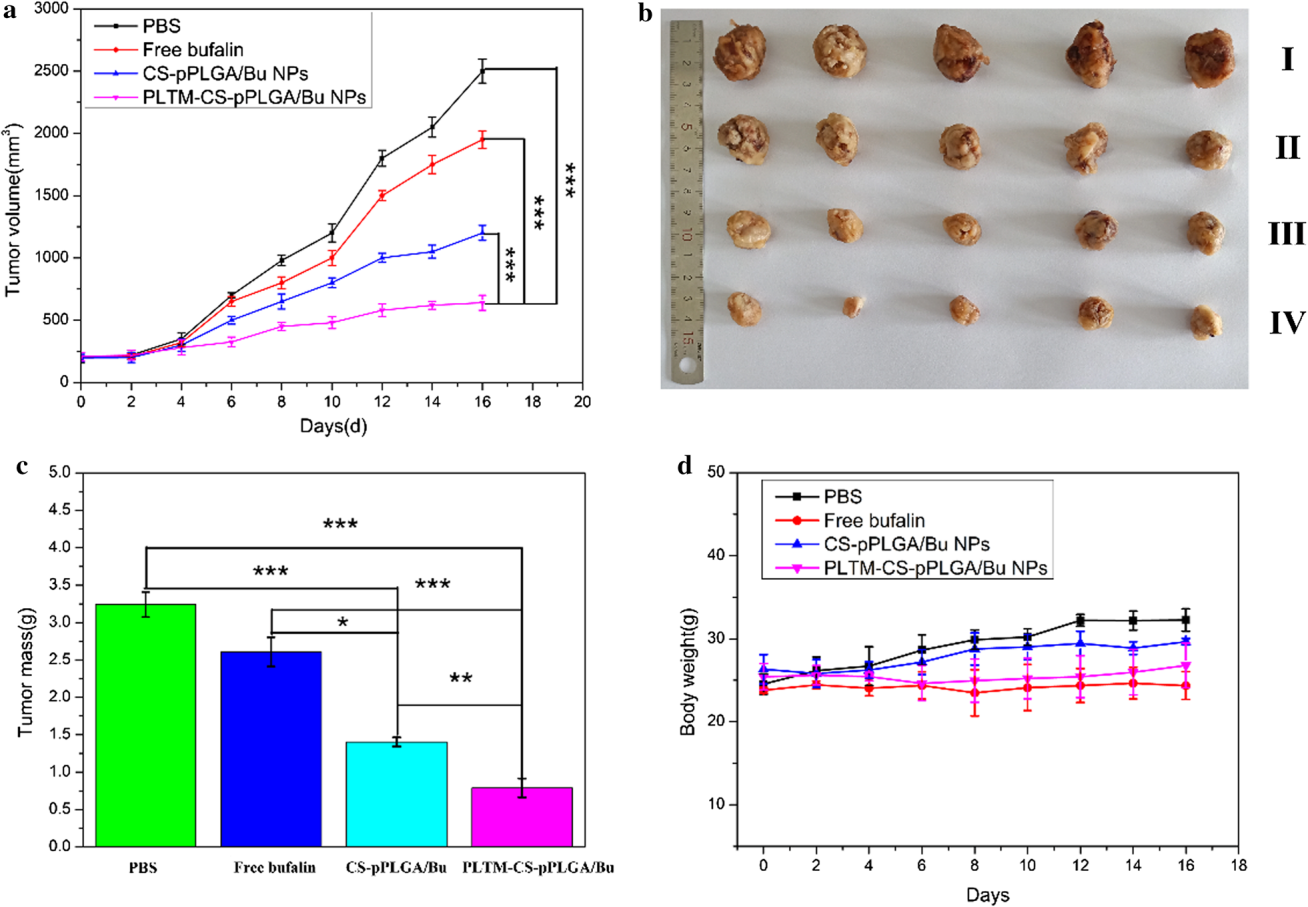
In vivo antitumor efficacy of different bufalin formulations in H22-tumor bearing ICR mice. **a** H22 tumor growth curves after intravenous injection the formulations. Error bars indicate SD (n = 5). **b** Representative images of tumors isolated after treatment for 16 days (1: PBS; 2: free bufalin; 3: CS-pPLGA/Bu NPs; 4: PLTM-CS-pPLGA/Bu NPs). **c** Mean weights of the H22 tumors isolated on day 16. **d** Body weight changes over the 16 days of the experiment (mean ± SD, n = 5)

The in vivo antitumor efficacy was then assessed using H22 tumor-bearing ICR mice. The results (Fig. 9) revealed that continuous tumor progression was witnessed for the mice treated with PBS and free bufalin, likely owing to the insufficient tumor retention of free bufalin. This manifested in a rapid growth in tumor volume over the 16 days of the experiment (Fig. 9a–c). For mice treated with CS-pPLGA/Bu NPs, tumor growth was significantly inhibited, and for animals receiving PLTM-CS-pPLGA/Bu NPs, the tumors showed the slowest growth and smallest volume at the end of the treatment. When the mice were treated with PBS, a continuous increase in body weight was noted (Fig. 9d) as a result of the increasing tumor size. The mice treated with other bufalin formulations showed no such increase, with no noticeable change in body weight. This indicates that the drug and nanoparticles had low systemic toxicity.

**Fig. 10:**
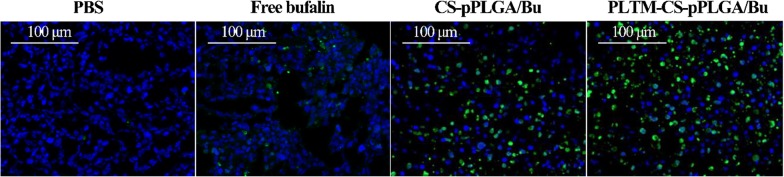
TUNEL-stained images of tumor slices taken from the various treatment groups

In addition, in situ TUNEL assay results (Fig. 10) showed no green coloration in the tumors of mice treated with PBS, indicating the cells all to be viable. Some green-colored cells are noticeable with all the bufalin treatments, indicative of apoptosis. The highest levels of apoptosis were seen in the tumors resected from mice treated with PLTM-CS-pPLGA/Bu NPs, as is clear from the increased number of green-colored cells. Therefore, the PLTM cloaked drug-loaded nanoparticles induce apoptosis more effectively than either free bufalin or the naked NPs.

Off-target toxicity is always a concern with cancer therapies. Histological analyses of major organs post-sacrifice were thus conducted (Additional file 1: Figure S10). The images obtained from untreated mice and the PLTM-CS-pPLGA/Bu NPs are very similar, and hence it is clear that the NPs do not cause any significant systemic toxicity.

### Biosafety in vivo

To investigate the feasibility of the PLTM biomimetic CS-pPLGA NPs for clinical translation, we further evaluated the biosafety in vivo. H22-tumor bearing ICR mice were i.v. injected with PBS or PLTM-CS-pPLGA NPs. No death or discomfort was observed during the experiment. The mice treated with PLTM-CS-pPLGA NPs showed no significant tumor volume difference compared to the control group (Additional file 1: Figure S11a), indicating that the PLTM biomimetic NPs can’t cause tumor growth inhibition. Histology analysis of the liver and kidney (Additional file 1: Figure S11b) showed no inflammation or lesions. The sections from NP-treated mice are very similar to the negative control group, indicating that the NPs do not cause systemic toxicity.

As shown in Additional file 1: Figure S11c and 11d, hepatic function markers (ALT and AST) and renal function markers (CREA and BUN) were determined to be normal and again very similar to the control group, suggesting no obvious hepatic or kidney disorders were induced by the PLTM-CS-pPLGA NPs in vivo. Collectively, these results demonstrated that the repeated administration of the PLTM cloaked NPs has a low risk of off-target toxicity.

Compared to other PLGA-based nanoscale delivery systems, the PLTM-modified materials prepared in this work have a number of advantages. For example, Xu et al. [36] aimed to improve the biocompatibility of PLGA by modifying it with poly(ethylene glycol) (PEG); they prepared a monomethoxy poly(ethylene glycol)-b-PLGA-b-poly(l-glutamic acid) (mPEG-PLGA-PGlu) nanoparticles. Although the PEG segment improved the formulation’s biocompatibility, the synthetic procedure was complicated and did not allow for any active targeting. Other biomimetic delivery systems, for example red blood cell membrane (RBCM) cloaked nanoparticles [17], have excellent biocompatibility, low immunogenicity and prolonged circulation times in vivo and have been suggested to have enhanced anti-tumor efficacy. However, the lack of active targeting to tumor cells by the RBCM limits its use for preparing biomimetic cancer therapeutics. The use of the PLTM overcomes this issue, and provides enhanced efficacy through specific binding of P-selectin to the CD44 receptors overexpressed on the surface of H22 cells.

## Conclusions

In this study, we developed platelet membrane (PLTM)-functionalized porous nanoparticles (NPs) which permit active-targeting mediated delivery of bufalin to tumor sites. These nanoparticles comprised a PLGA-chitosan conjugate, and were able to achieve a bufalin loading of ~ 8.5%. The PLTM coating led to particles providing pH-dependent sustained drug release over 2 days. The carrier was found to be biocompatible with H22 cells, while the drug-loaded analogue could effectively kill cancer cells in vitro. A high level of uptake of PLTM-coated NPs by cancer cells was observed by confocal microscopy and flow cytometry analysis. In vivo imaging showed accumulation of the NPs in the tumor site, demonstrating their active targeting ability as a result of targeted binding of P-selectin to the CD44 receptors overexpressed on the surface of H22 cells. The PLTM-coated NPs were also found to be effective in reducing tumor growth in vivo, and did not cause off-target effects on the major organs. Therefore, the novel PLTM biomimetic formulations reported in this work have significant potential as anti-cancer drug carriers. Furthermore, platelets have the ability to selective adhere to injured blood vessels [37], so the PLTM-coated material could also be used to deliver vascular disrupting agents or combined with photothermal therapy [38] to damage tumor blood vessels, and thus achieve enhanced anti-tumor activity.

## Additional file

**Additional file 1: Figure S1.** The ^1^H-NMR spectra of (A) CS, (B) PLGA, and (C) CS-PLGA. **Figure S2.** Zeta potential values of CS, PLGA, and CS-PLGA in aqueous media at pH 5.0, 7.4, and 8.6. **Figure S3.** N_2_ adsorption/desorption isotherms of CS-pPLGA NPs and the corresponding pore-size distribution curves (inset). **Figure S4.** (A) A digital photograph taken during the isolation of PLTs from plasma, and bright field microscopy images of (B) PLTs and (C) RBCs. **Figure S5.** Zeta potential values of PLTM, CS-pPLGA and PLTM-CS-pPLGA NPs at pH=7.4. **Figure S6.** Western blotting results for key platelet membrane protein bands. **Figure S7.** CLSM images of RAW 264.7 cells following 2 h incubation with PLTM-CS-pPLGA NPs. Scale bar: 20 μm. **Figure S8.** Microscope images of RBCs incubated with (a) PLTM-CS-pPLGA NPs, (b) PBS (negative control), (c) Triton X-100 (positive control), and corresponding pictures after centrifugation (5000 rpm, 10 min). RBCs precipitate indicated no hemolysis. **Figure S9**. (a) Time-dependent *in vivo* fluorescence imaging of Cy5.5 labeled CS-pPLGA NPs in H22 tumor-bearing ICR mice. (b) *Ex vivo* fluorescence imaging of the excised tumors and normal organs at 6 h post-injection. (c) ROI analysis of fluorescent intensities from the tumor and major organs. Error bars indicate S.D. (n = 3). **Figure S10.** H&E stained images of the major organs collected from (I) untreated mice and (II) mice treated for 16 days with PLTM-CS-pPLGA/Bu NPs. **Figure S11.** (a) H22-tumor bearing mice tumor mass changes over 16 days (mean ± S.D., n = 5); (b) histological analyses of the liver and kidney of mice following 16 days’ treatment with PBS and PLTM-CS-pPLGA NPs (scale bar: 50 μm); (c) and (d) blood biochemical analyses of the mice treated with PBS or the PLTM-CS-pPLGA NPs. **Table S1.** Encapsulation efficiency and loading content of NPs prepared under different CS-PLGA/Bu (w/w) ratio (mean ± SD, n = 3). **Table S2.** Hemocompatibility data. Each number indicating the average of three times spectroscopic measurements.

## Data Availability

All data generated or analyzed during this study are included in this published article.

## Abbreviations

DDSs: drug-delivery systems
NPs: nanoparticles
CS: Chitosan oligosaccharide
PLGA: poly(lactic-co-glycolic acid)
Bu: bufalin
PLTM: platelet membrane
PEG: poly(ethylene glycol)
TPGS: vitamin E polyethylene glycol succinate
FITC: fluoresceinisothiocyanate
Cy5.5–COOH: cyanine-5,5–carboxylic acid

## References

[1] Lin LS, Song J, Song L, Ke K, Liu Y, Zhou Z, Shen Z, Li J, Yang Z, Tang W (2018). Simultaneous fenton-like ion delivery and glutathione depletion by MnO_2_-based nanoagent enhances chemodynamic therapy. Angew Chem Int Ed Engl.

[2] Li J, Zou S, Gao J, Liang J, Zhou H, Liang L, Wu W (2017). Block copolymer conjugated Au-coated Fe_3_O_4_ nanoparticles as vectors for enhancing colloidal stability and cellular uptake. J Nanobiotechnol.

[3] Rizzitelli S, Giustetto P, Faletto D, Delli Castelli D, Aime S, Terreno E (2016). The release of doxorubicin from liposomes monitored by MRI and triggered by a combination of US stimuli led to a complete tumor regression in a breast cancer mouse model. J Control Release.

[4] Hinger D, Gräfe S, Navarro F, Spingler B, Pandiarajan D, Walt H, Couffin AC, Maake C (2016). Lipid nanoemulsions and liposomes improve photodynamic treatment efficacy and tolerance in CAL-33 tumor bearing nude mice. J Nanobiotechnol.

[5] Peppas NA, Hilt JZ, Khademhosseini A, Langer R (2006). Hydrogels in biology and medicine: from molecular principles to bionanotechnology. Adv Mater.

[6] Chao Y, Xu L, Liang C, Feng L, Xu J, Dong Z, Tian L, Yi X, Yang K, Liu Z (2018). Combined local immunostimulatory radioisotope therapy and systemic immune checkpoint blockade imparts potent antitumour responses. Nat Biomed Eng.

[7] Sun W, Zhang J, Zhang C, Wang P, Peng C, Shen M, Shi X (2018). Construction of hybrid alginate nanogels loaded with manganese oxide nanoparticles for enhanced tumor magnetic resonance imaging. ACS Macro Lett.

[8] Wu J, Deng C, Meng F, Zhang J, Sun H, Zhong Z (2017). Hyaluronic acid coated PLGA nanoparticulate docetaxel effectively targets and suppresses orthotopic human lung cancer. J Control Release.

[9] Hu D, Chen L, Qu Y, Peng J, Chu B, Shi K, Hao Y, Zhong L, Wang M, Qian Z (2018). Oxygen-generating hybrid polymeric nanoparticles with encapsulated doxorubicin and chlorin e6 for trimodal imaging-guided combined chemo-photodynamic therapy. Theranostics.

[10] Senevirathne SA, Washington KE, Biewer MC, Stefan MC (2016). PEG based anti-cancer drug conjugated prodrug micelles for the delivery of anti-cancer agents. J Mater Chem B.

[11] Wang Y, Chen L, Tan L, Zhao Q, Luo F, Wei Y, Qian Z (2014). PEG-PCL based micelle hydrogels as oral docetaxel delivery systems for breast cancer therapy. Biomaterials.

[12] Li B, Li W, Perez-Aguilar JM, Zhou R (2017). Mild binding of protein to C_2_N monolayer reveals its suitable biocompatibility. Small.

[13] Vader P, van der Aa LJ, Engbersen JF, Storm G, Schiffelers RM (2012). Physicochemical and biological evaluation of siRNA polyplexes based on PEGylated Poly(amido amine)s. Pharm Res.

[14] Gratton SEA, Ropp PA, Pohlhaus PD, Luft JC, Madden VJ, Napier ME, Desimone JM (2008). The effect of particle design on cellular internalization pathways. Proc Natl Acad Sci USA.

[15] Hu CM, Fang RH, Wang KC, Luk BT, Thamphiwatana S, Dehaini D, Nguyen P, Angsantikul P, Wen CH, Kroll AV (2015). Nanoparticle biointerfacing by platelet membrane cloaking. Nature.

[16] Zolnik BS, Gonzalez-Fernandez A, Sadrieh N, Dobrovolskaia MA (2010). Nanoparticles and the immune system. Endocrinology.

[17] Han X, Wang C, Liu Z (2018). Red blood cells as smart delivery systems. Bioconjug Chem.

[18] Zhu DM, Wu L, Suo M, Gao S, Xie W, Zan MH, Liu A, Chen B, Wu WT, Ji LW (2018). Engineered red blood cells for capturing circulating tumor cells with high performance. Nanoscale.

[19] Gao M, Liang C, Song X, Chen Q, Jin Q, Wang C, Liu Z (2017). Erythrocyte-membrane-enveloped perfluorocarbon as nanoscale artificial red blood cells to relieve tumor hypoxia and enhance cancer radiotherapy. Adv Mater.

[20] Martinez JO, Molinaro R, Hartman KA, Boada C, Sukhovershin R, De Rosa E, Kirui D, Zhang S, Evangelopoulos M, Carter AM (2018). Biomimetic nanoparticles with enhanced affinity towards activated endothelium as versatile tools for theranostic drug delivery. Theranostics.

[21] Rao L, Bu L-L, Meng Q-F, Cai B, Deng W-W, Li A, Li K, Guo S-S, Zhang W-F, Liu W (2017). Antitumor platelet-mimicking magnetic nanoparticles. Adv Funct Mater.

[22] Schurch CM, Forster S, Bruhl F, Yang SH, Felley-Bosco E, Hewer E (2017). The “don’t eat me” signal CD47 is a novel diagnostic biomarker and potential therapeutic target for diffuse malignant mesothelioma. Oncoimmunology.

[23] Hu Q, Sun W, Qian C, Wang C, Bomba HN, Gu Z (2015). Anticancer platelet-mimicking nanovehicles. Adv Mater.

[24] Takai N, Ueda T, Nishida M, Nasu K, Narahara H (2008). Bufalin induces growth inhibition, cell cycle arrest and apoptosis in human endometrial and ovarian cancer cells. Int J Mol Med.

[25] Meng Z, Yang P, Shen Y, Bei W, Zhang Y, Ge Y, Newman RA, Cohen L, Liu L, Thornton B (2009). Pilot study of huachansu in patients with hepatocellular carcinoma, nonsmall-cell lung cancer, or pancreatic cancer. Cancer.

[26] Newman RA, Yang P, Pawlus AD, Block KI (2008). Cardiac glycosides as novel cancer therapeutic agents. Mol Interv.

[27] Yu CH, Kan SF, Pu HF, Jea Chien E, Wang PS (2008). Apoptotic signaling in bufalin- and cinobufagin-treated androgen-dependent and -independent human prostate cancer cells. Cancer Sci.

[28] Park W, Kim D, Kang HC, Bae YH, Na K (2012). Multi-arm histidine copolymer for controlled release of insulin from poly(lactide-co-glycolide) microsphere. Biomaterials.

[29] Zhu H, Chen H, Zeng X, Wang Z, Zhang X, Wu Y, Gao Y, Zhang J, Liu K, Liu R (2014). Co-delivery of chemotherapeutic drugs with vitamin E TPGS by porous PLGA nanoparticles for enhanced chemotherapy against multi-drug resistance. Biomaterials.

[30] Wu J, Williams GR, Branford-White C, Li H, Li Y, Zhu LM (2016). Liraglutide-loaded poly(lactic-co-glycolic acid) microspheres: preparation and in vivo evaluation. Eur J Pharm Sci.

[31] Roointan A, Farzanfar J, Mohammadi-Samani S, Behzad-Behbahani A, Farjadian F (2018). Smart pH responsive drug delivery system based on poly(HEMA-co-DMAEMA) nanohydrogel. Int J Pharm.

[32] Kim Y, Lobatto ME, Kawahara T, Lee CB, Mieszawska AJ, Sanchezgaytan BL, Fay F, Senders ML, Calcagno C, Becraft J (2014). Probing nanoparticle translocation across the permeable endothelium in experimental atherosclerosis. Proc Natl Acad Sci USA.

[33] Luk BT, Hu CM, Fang RH, Dehaini D, Carpenter C, Gao W, Zhang L (2014). Interfacial interactions between natural RBC membranes and synthetic polymeric nanoparticles. Nanoscale.

[34] Taghavi S, Ramezani M, Alibolandi M, Abnous K, Taghdisi SM (2017). Chitosan-modified PLGA nanoparticles tagged with 5TR1 aptamer for in vivo tumor-targeted drug delivery. Cancer Lett.

[35] Yu S-R, Zhang X-P, He Z-M, Liu Y-H, Liu Z-H (2004). Effects of Ce on the short-term biocompatibility of Ti–Fe–Mo–Mn–Nb–Zr alloy for dental materials. J Mater Sci Mater Med.

[36] Xu H, Yang D, Cai C, Gou J, Zhang Y, Wang L, Zhong H, Tang X (2015). Dual-responsive mPEG-PLGA-PGlu hybrid-core nanoparticles with a high drug loading to reverse the multidrug resistance of breast cancer: an in vitro and in vivo evaluation. Acta Biomater.

[37] Song Y, Huang Z, Liu X, Pang Z, Chen J, Yang H, Zhang N, Cao Z, Liu M, Cao J (2018). Platelet membrane-coated nanoparticle-mediated targeting delivery of rapamycin blocks atherosclerotic plaque development and stabilizes plaque in apolipoprotein E-deficient (ApoE(−/−)) mice. Nanomedicine.

[38] Zhang F, Cao J, Chen X, Yang K, Zhu L, Fu G, Huang X, Chen X (2015). Noninvasive dynamic imaging of tumor early response to nanoparticle-mediated photothermal therapy. Theranostics.

