# Supplementary material for: Platelet-membrane-biomimetic nanoparticles for targeted antitumor drug delivery
Source: J Nanobiotechnology. 2019 May 13;17:60. doi: 10.1186/s12951-019-0494-y (PMC6513513; doi:10.1186/s12951-019-0494-y)
Supplement: Supplementary file 1 — Additional file 1: Figure S1. The 1H-NMR spectra of (A) CS, (B) PLGA, and (C) CS-PLGA. Figure S2. Zeta potential values of CS, PLGA, and CS-PLGA in aqueous media at pH 5.0, 7.4, and 8.6. Figure S3. N2 adsorption/desorption isotherms of CS-pPLGA NPs and the corresponding pore-size distribution curves (inset). Figure S4. (A) A digital photograph taken during the isolation of PLTs from plasma, and bright field microscopy images of (B) PLTs and (C) RBCs. Figure S5. Zeta potential values of PLTM, CS-pPLGA and PLTM-CS-pPLGA NPs at pH=7.4. Figure S6. Western blotting results for key platelet membrane protein bands. Figure S7. CLSM images of RAW 264.7 cells following 2 h incubation with PLTM-CS-pPLGA NPs. Scale bar: 20 μm. Figure S8. Microscope images of RBCs incubated with (a) PLTM-CS-pPLGA NPs, (b) PBS (negative control), (c) Triton X-100 (positive control), and corresponding pictures after centrifugation (5000 rpm, 10 min). RBCs precipitate indicated no hemolysis. Figure S9. (a) Time-dependent in vivo fluorescence imaging of Cy5.5 labeled CS-pPLGA NPs in H22 tumor-bearing ICR mice. (b) Ex vivo fluorescence imaging of the excised tumors and normal organs at 6 h post-injection. (c) ROI analysis of fluorescent intensities from the tumor and major organs. Error bars indicate S.D. (n = 3). Figure S10. H&E stained images of the major organs collected from (I) untreated mice and (II) mice treated for 16 days with PLTM-CS-pPLGA/Bu NPs. Figure S11. (a) H22-tumor bearing mice tumor mass changes over 16 days (mean ± S.D., n = 5); (b) histological analyses of the liver and kidney of mice following 16 days’ treatment with PBS and PLTM-CS-pPLGA NPs (scale bar: 50 μm); (c) and (d) blood biochemical analyses of the mice treated with PBS or the PLTM-CS-pPLGA NPs. Table S1. Encapsulation efficiency and loading content of NPs prepared under different CS-PLGA/Bu (w/w) ratio (mean ± SD, n = 3). Table S2. Hemocompatibility data. Each number indicating the average of three times spectroscopic [file 12951_2019_494_MOESM1_ESM.docx]

**Additional Information for:**

**Platelet-membrane-biomimetic nanoparticles for targeted antitumor drug delivery**

Haijun Wang^1^, Junzi Wu^2*^, Gareth R. Williams^3^, Qing Fan^4^, Shiwei Niu^1^, Jianrong Wu^1^, Xiaotian Xie^1^, Li-Min Zhu^1*^

^1^ College of Chemistry, Chemical Engineering and Biotechnology, Donghua University, Shanghai, 201620, China

^2^ College of Basic Medicine, Yunnan University of Traditional Chinese Medicine, Kunming, 650500, China

^3^ UCL School of Pharmacy, University College London, 29-39 Brunswick Square, London, WC1N 1AX, UK

^4^ Department of Pharmacy, Shandong Cancer Hospital Affiliated to Shandong University, Shandong Academy of Medical Science, Jinan, 250117, China

* Corresponding authors: E-mail: [lzhu@dhu.edu.cn](mailto:lzhu@dhu.edu.cn) (Li-Min Zhu); [beached@126.com](mailto:beached@126.com%20) (Junzi Wu)

**The file includes**

Figure S1-S11, Table S1, Table S2


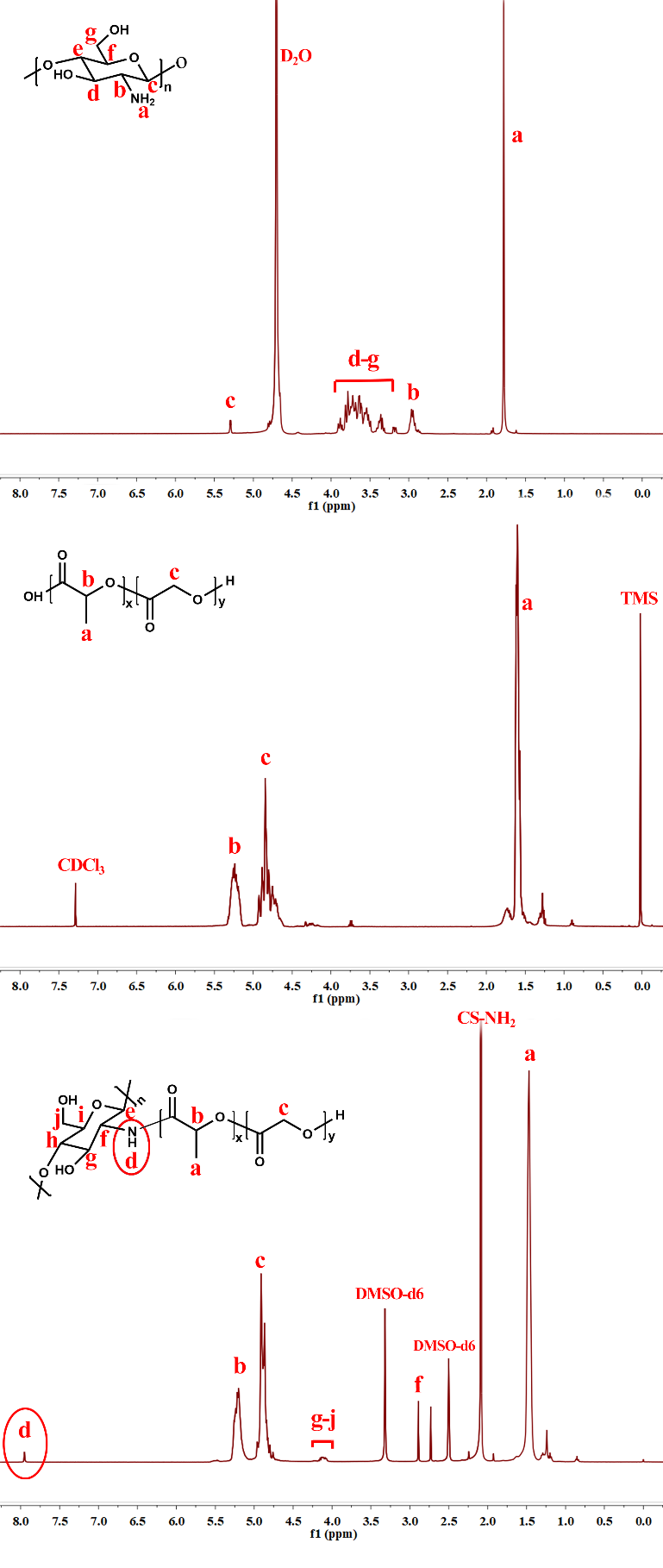


**Figure S1.** The ^1^H-NMR spectra of (A) CS, (B) PLGA, and (C) CS-PLGA.


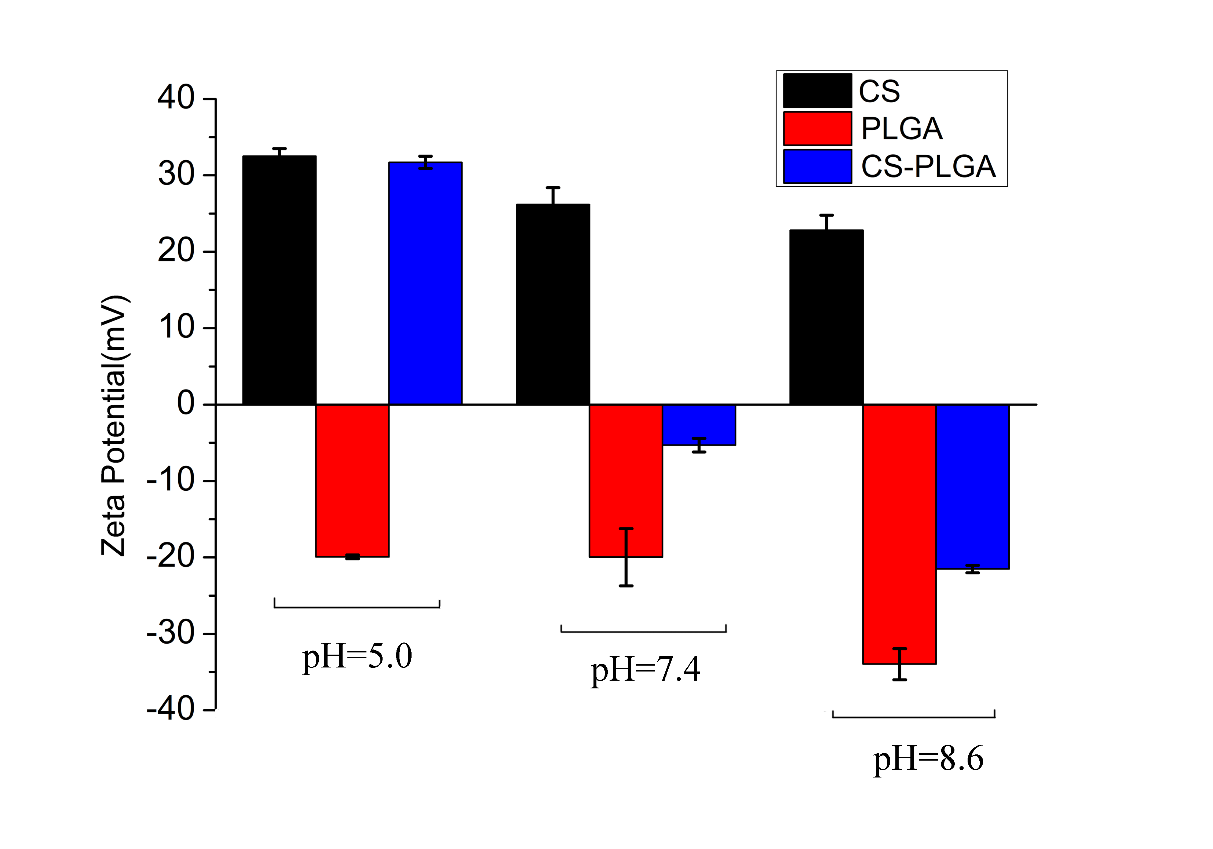


**Figure S2.** Zeta potential values of CS, PLGA, and CS-PLGA in aqueous media at pH 5.0, 7.4, and 8.6.

**
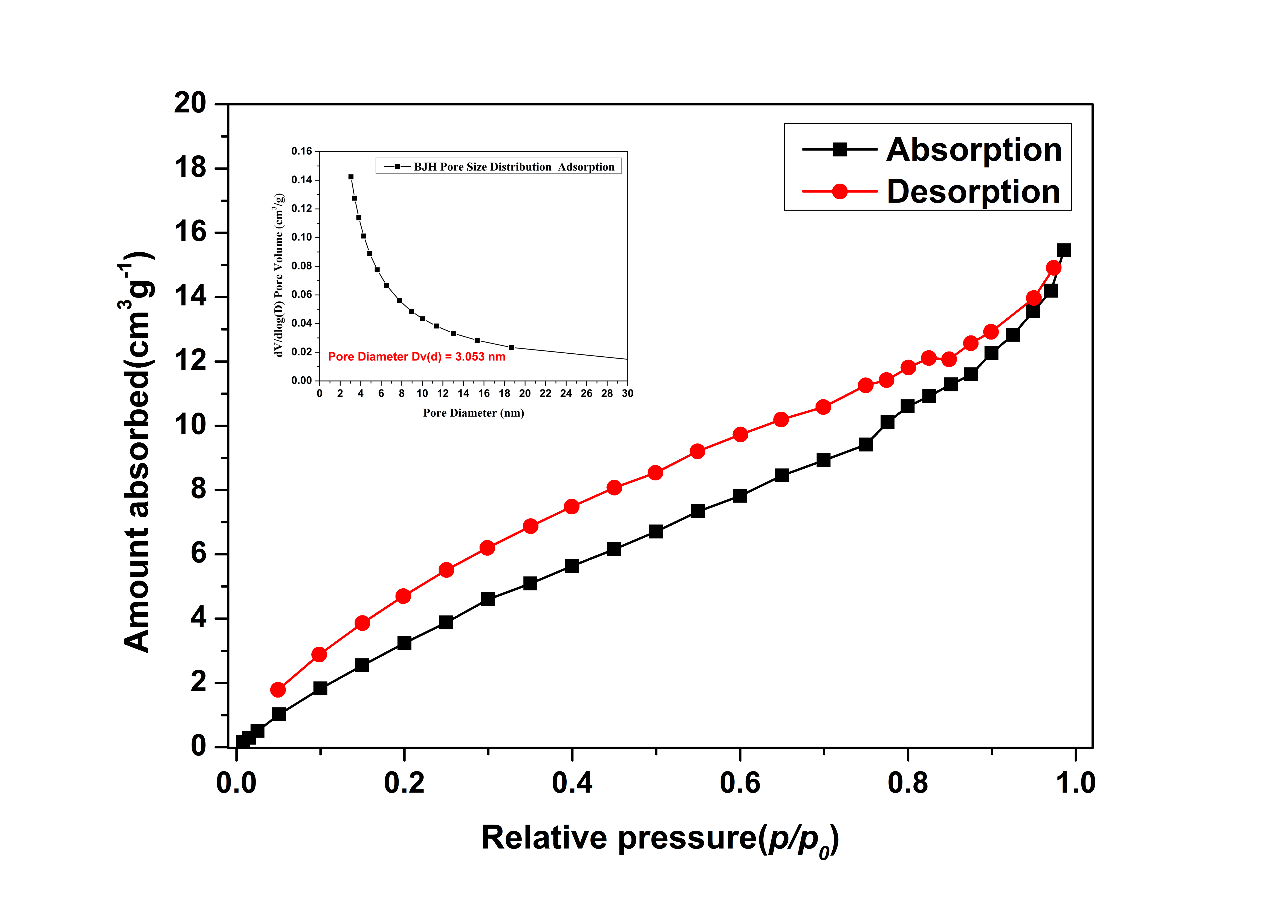
Figure S3.** N_2_ adsorption/desorption isotherms of CS-pPLGA NPs and the corresponding pore-size distribution curves (inset).

**
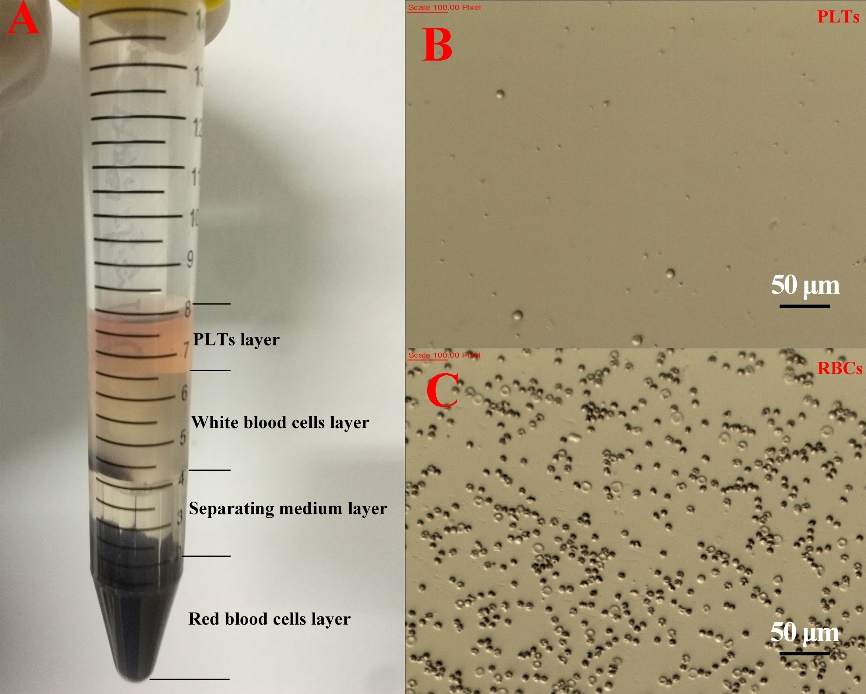
**

**Figure S4.** (A) A digital photograph taken during the isolation of PLTs from plasma, and bright field microscopy images of (B) PLTs and (C) RBCs.

**
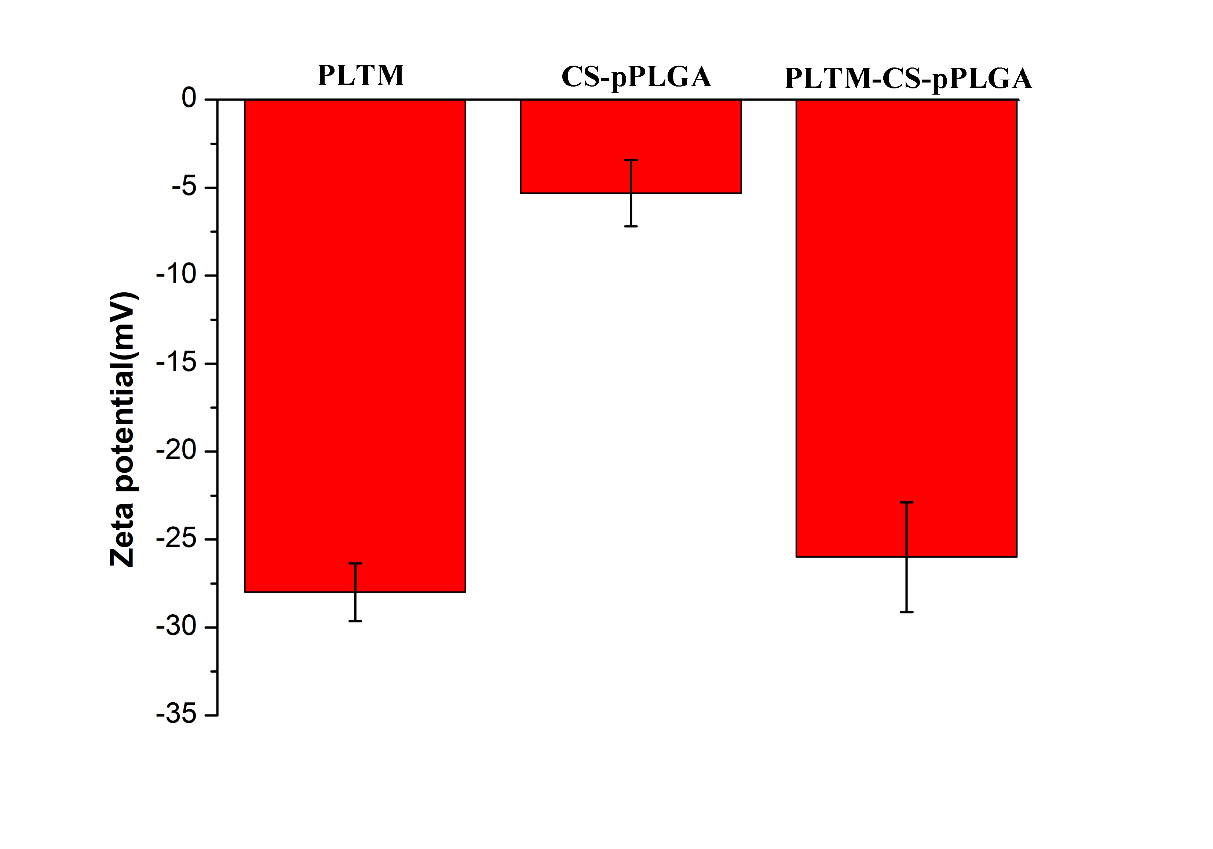
 Figure S5.** Zeta potential values of PLTM, CS-pPLGA and PLTM-CS-pPLGA NPs at pH=7.4.

**
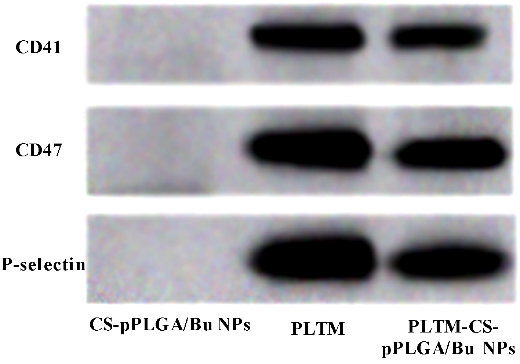
**

**Figure S6.** Western blotting results for key platelet membrane protein bands.

**
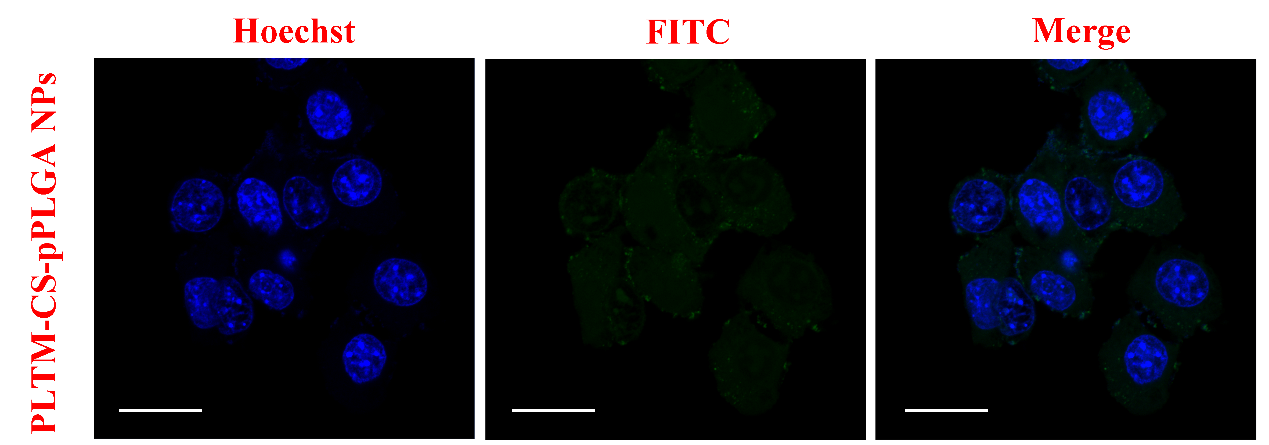
**

**Figure S7.** CLSM images of RAW 264.7 cells following 2 h incubation with PLTM-CS-pPLGA NPs. Scale bar: 20 μm.

**
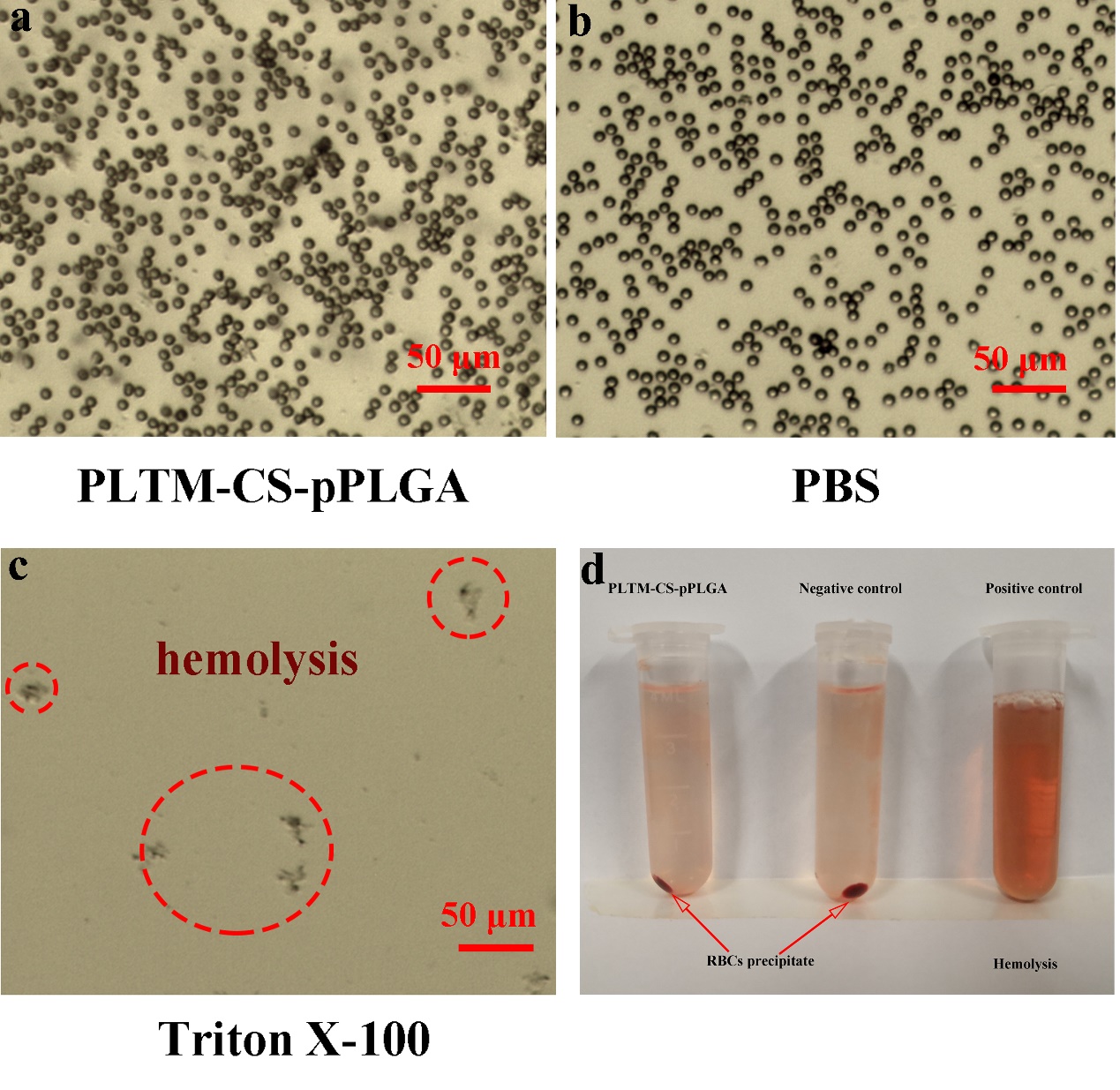
**

**Figure S8.** Microscope images of RBCs incubated with (a) PLTM-CS-pPLGA NPs, (b) PBS (negative control), (c) Triton X-100 (positive control), and corresponding pictures after centrifugation (5000 rpm, 10 min). RBCs precipitate indicated no hemolysis.

**
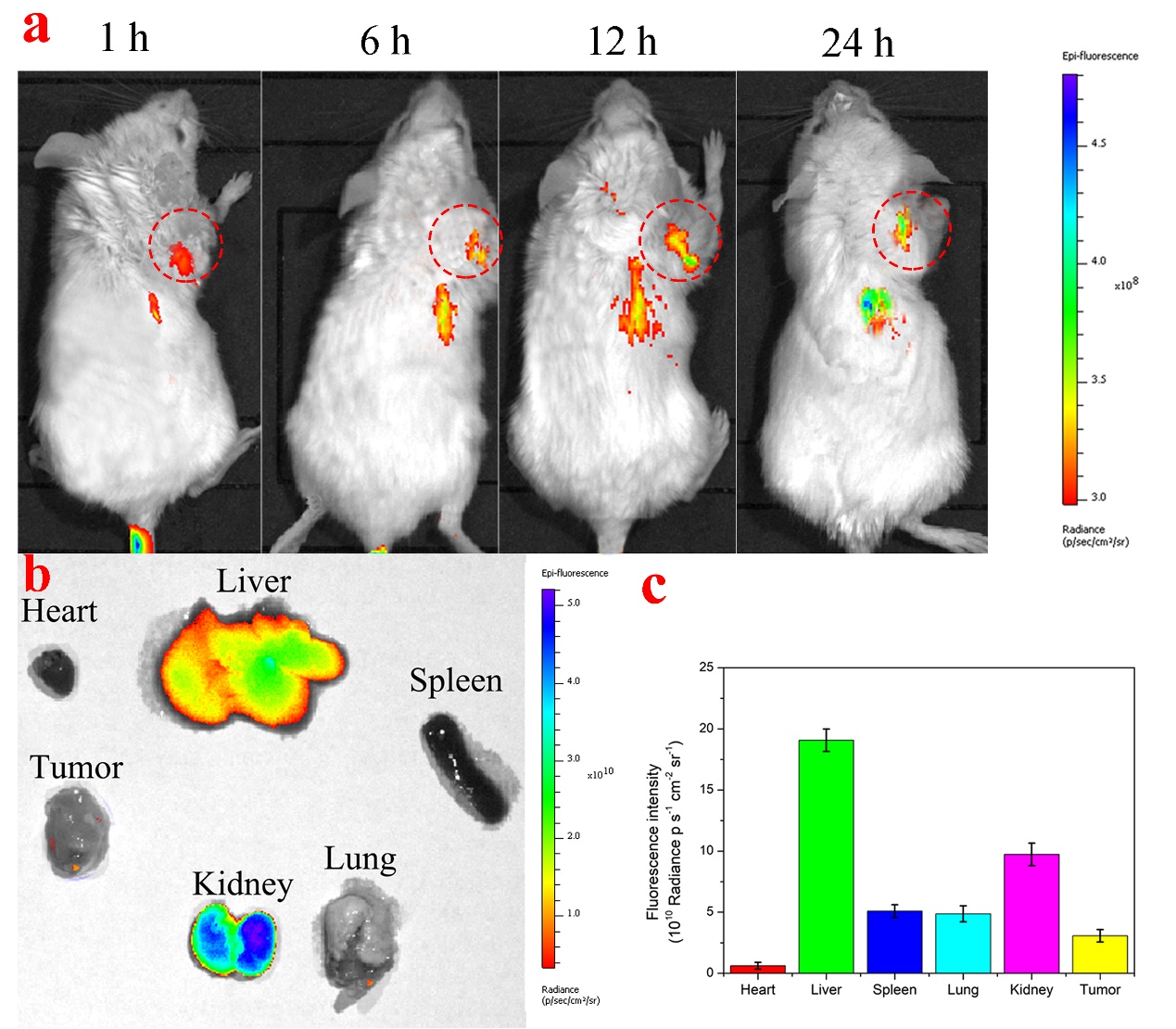
**

**Figure S9**. (a) Time-dependent *in vivo* fluorescence imaging of Cy5.5 labeled CS-pPLGA NPs in H22 tumor-bearing ICR mice. (b) *Ex vivo* fluorescence imaging of the excised tumors and normal organs at 6 h post-injection. (c) ROI analysis of fluorescent intensities from the tumor and major organs. Error bars indicate S.D. (n = 3).

**
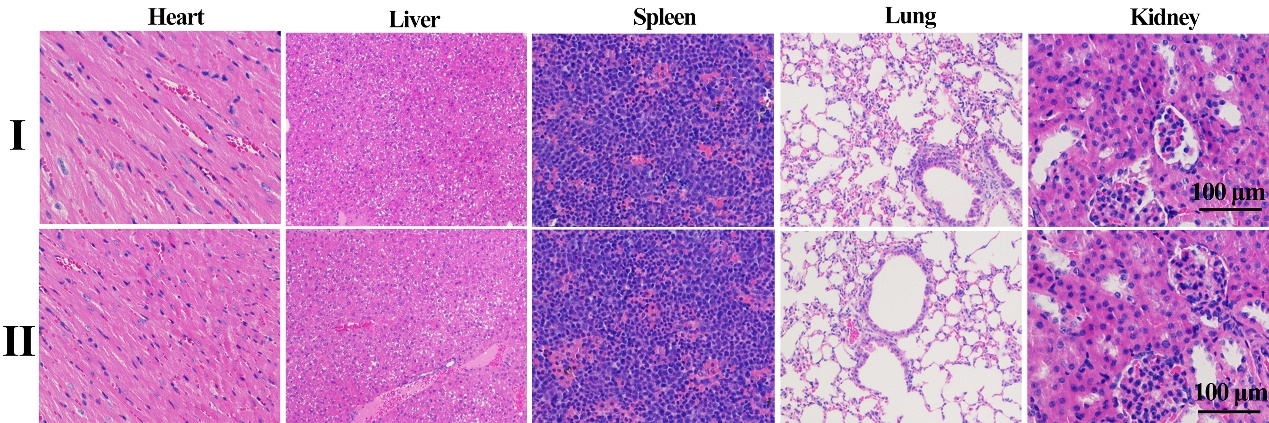
**

**Figure S10.** H&E stained images of the major organs collected from (I) untreated mice and (II) mice treated for 16 days with PLTM-CS-pPLGA/Bu NPs.


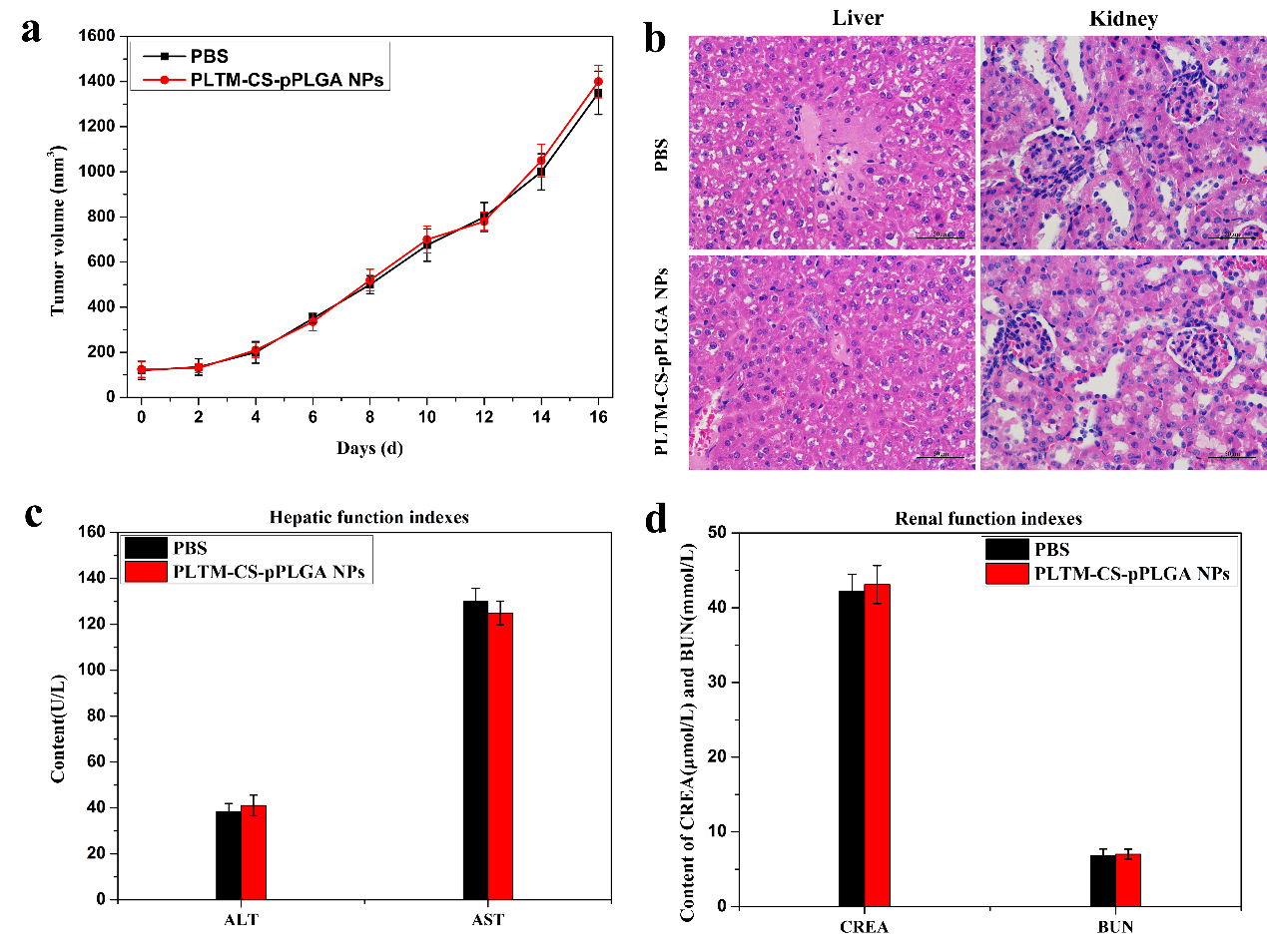


**Figure S11.** (a) H22-tumor bearing mice tumor mass changes over 16 days (mean ± S.D., n = 5); (b) histological analyses of the liver and kidney of mice following 16 days’ treatment with PBS and PLTM-CS-pPLGA NPs (scale bar: 50 μm); (c) and (d) blood biochemical analyses of the mice treated with PBS or the PLTM-CS-pPLGA NPs.

**Table S1**: Encapsulation efficiency and loading content of NPs prepared under different CS-PLGA/Bu (w/w) ratio (mean ± SD, n = 3).

| Ratio | LC (%) | EE (%) |
| --- | --- | --- |
| 1:20 | 4.6 ± 1.3 | 96.4 ± 4.2 |
| 1:10 | 8.5 ±1.9 | 92.8 ± 3.5 |
| 1:5 | 11.5 ± 2.1 | 65.4 ± 4.2 |

**Table S2.** Hemocompatibility data. Each number indicating the average of three times spectroscopic measurements.

| Treatment | OD value (average) | Hemolysis (%) |
| --- | --- | --- |
| Positive control | 0.3250 | 100 |
| Negative control | 0.0703 | 0 |
| PLTM-CS-pPLGA | 0.0828 | 3.85 |
